# A high-affinity RBD-targeting nanobody improves fusion partner’s potency against SARS-CoV-2

**DOI:** 10.1371/journal.ppat.1009328

**Published:** 2021-03-03

**Authors:** Hebang Yao, Hongmin Cai, Tingting Li, Bingjie Zhou, Wenming Qin, Dimitri Lavillette, Dianfan Li

**Affiliations:** 1 CAS Center for Excellence in Molecular Cell Science, Shanghai Institute of Biochemistry and Cell Biology, Chinese Academy of Sciences, Shanghai, China; 2 University of Chinese Academy of Sciences, Beijing, China; 3 CAS Key Laboratory of Molecular Virology & Immunology, Institut Pasteur of Shanghai, Chinese Academy of Sciences, Shanghai, China; 4 National Facility for Protein Science in Shanghai, Shanghai Advanced Research Institute (Zhangjiang Laboratory), Chinese Academy of Sciences, Shanghai, China; 5 Pasteurien College, Soochow University, Jiangsu, China; Erasmus Medical Center, NETHERLANDS

## Abstract

A key step to the SARS-CoV-2 infection is the attachment of its Spike receptor-binding domain (S RBD) to the host receptor ACE2. Considerable research has been devoted to the development of neutralizing antibodies, including llama-derived single-chain nanobodies, to target the receptor-binding motif (RBM) and to block ACE2-RBD binding. Simple and effective strategies to increase potency are desirable for such studies when antibodies are only modestly effective. Here, we identify and characterize a high-affinity synthetic nanobody (sybody, SR31) as a fusion partner to improve the potency of RBM-antibodies. Crystallographic studies reveal that SR31 binds to RBD at a conserved and ‘greasy’ site distal to RBM. Although SR31 distorts RBD at the interface, it does not perturb the RBM conformation, hence displaying no neutralizing activities itself. However, fusing SR31 to two modestly neutralizing sybodies dramatically increases their affinity for RBD and neutralization activity against SARS-CoV-2 pseudovirus. Our work presents a tool protein and an efficient strategy to improve nanobody potency.

## Author summary

SARS-CoV-2 relies on the receptor-binding domain (RBD) of its envelope Spike protein to recognize and infect host cells. RBD is therefore an immunological hotspot to generate antibodies for therapeutic and detection purposes. Binding affinity is one of the key characteristics of such antibodies. Here, we report a single-chain antibody (nanobody, ~14 kDa) that binds RBD with nanomolar affinity. The nanobody, dubbed SR31, binds RBD at an epitope distal to the receptor-binding motif (RBM) which is the target of most neutralizing antibodies. SR31 can therefore bind RBD in addition to RBM-binders, and increase their affinity and potency by avidity effects when used as a fusion partner. Compared to other *in vitro* affinity maturation techniques such as library screening and structure-based design, the fusion strategy offers advantages in speed and simplicity. In addition, SR31, together with RBD-targeting nanobodies recognizing a wide spectrum of epitopes, provides a useful toolkit to probe epitopes of uncharacterized antibodies by competitive binding.

## Introduction

SARS-CoV-2, the pathogenic virus for COVID-19, has caused a global pandemic since its first report in early December 2019 in Wuhan, China [1], posing a grave crisis for the health and economic and social order. SARS-CoV-2 is heavily decorated by its surface Spike (S) [2, 3], a single-pass membrane protein that is key for the host-virus interactions. During the infection, S is cleaved by host proteases [4, 5], yielding the N-terminal S1 and the C-terminal S2 subunit. S1 binds to angiotensin-converting enzyme 2 (ACE2) [6–10] on the host cell membrane via its receptor-binding domain (RBD), causing conformational changes that trigger a secondary cleavage needed for the S2-mediated membrane fusion at the plasma membrane or in the endosome. Because of this essential role, RBD has been a hot spot for the development of therapeutic monoclonal antibodies (mAbs) and vaccine [11–28].

Llama-derived heavy chain-only antibodies (nanobodies) are attractive bio-therapeutics [29]. These small (~14 kDa) proteins are robust, straightforward to produce, and amenable to engineering such as mutation and fusion. Owing to their ultra-stability, nanobodies have been reported to survive nebulization, a feature that has been explored for the development of inhaled nanobodies to treat respiratory viral diseases [30, 31] which categorizes COVID-19. Owing to their high sequence similarities with human type 3 VH domains (VH3), nanobodies are considered to be poorly immunogenic in human [29]. For the same reason, they can be humanized with relative ease to reduce immunogenicity when needed. Therefore, nanobodies have been increasingly recognized as potential biotherapeutics. Examples of nanobody drugs include caplacizumab [32] for the treatment of acquired thrombotic thrombocytopenic purpura, and ozoralizumab and vobarilizumab that are in the clinical trials for rheumatoid arthritis [29, 33]. Recently, several groups have independently reported neutralizing nanobodies [22, 34–39] or single-chain VH antibodies [40] against SARS-CoV-2 with variable potencies.

We have recently reported several synthetic nanobodies (sybodies) that bind RBD with various affinity and neutralizing activity [35]. Affinity and neutralizing activity are very important characteristics for therapeutic antibodies, and they can be improved in several ways such as random mutagenesis [22, 36] and structure-based design. Previously, in the case of a modestly-neutralizing sybody (MR17), we have determined its structure and designed a single mutant that improves its potency by over 23 folds [35]. The rational design approach, while very effective, inevitably requires high-resolution structural information which is generally non-trivial to obtain. General applicable tools will be welcome.

Here, we report a strategy to increase sybody potency by biparatopic fusion with SR31, a sybody that binds RBD tightly with a *K*_D_ of 5.6 nM. Crystallographic studies reveal that SR31 engages the RBD at a conserved site that is distal to the RBM. As such, it does not neutralize SARS-CoV-2 but forms non-competing pairs with several other RBM-binders and increases their neutralization potency when conjugated. SR31 may be used as a general affinity-enhancer for both detection and therapeutic applications.

## Results

### A high-affinity RBD binder without neutralizing activity

Previously, we generated 99 sybodies from three highly diverse synthetic libraries by ribosome and phage display with *in vitro* selections against the SARS-CoV-2 RBD. Most of the RBD binders showed neutralizing activity. Interestingly, about 10 sybodies bind RBD but showed no neutralizing activities [35] even at 1 μM concentration.

**Fig 1:**
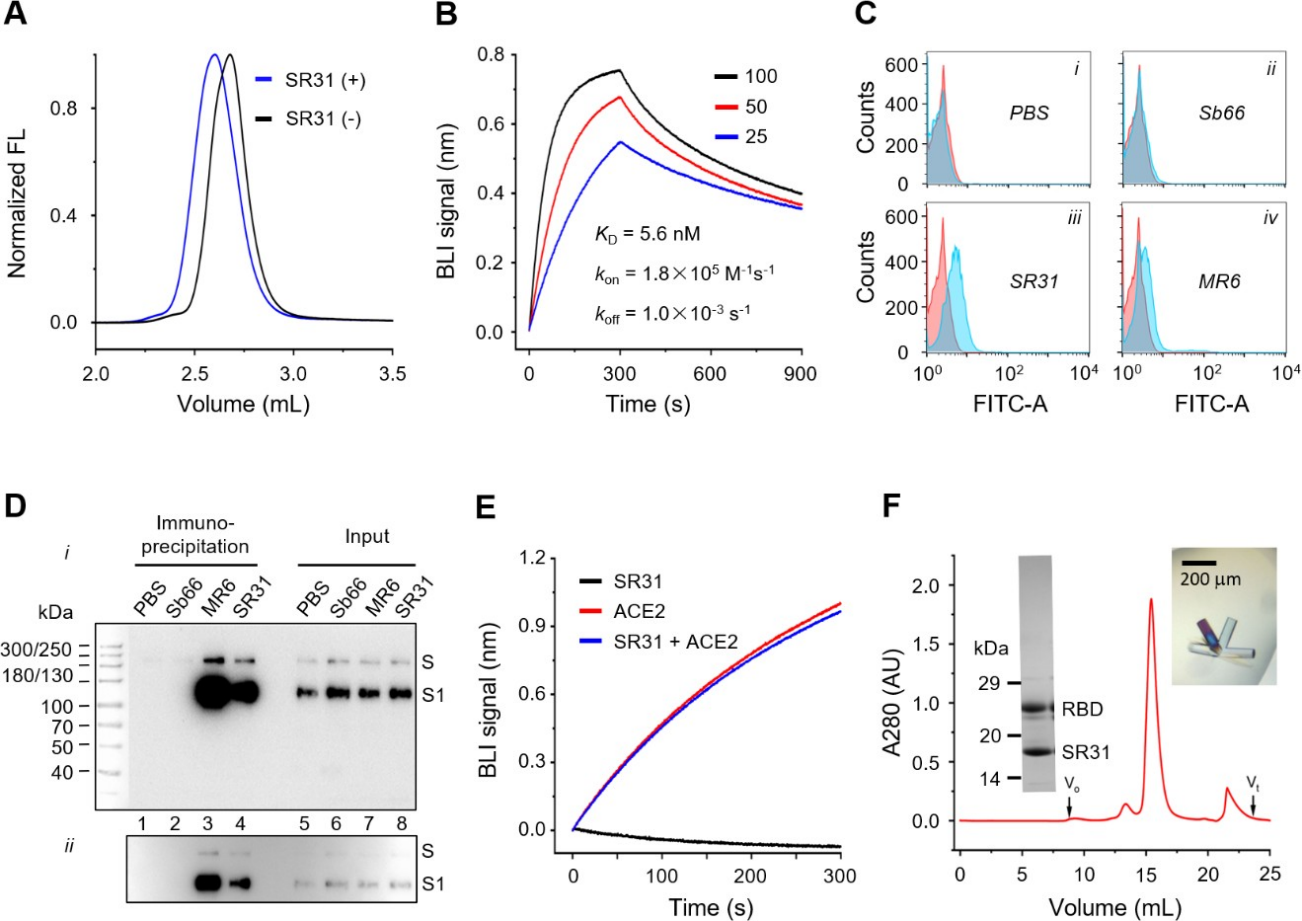
SR31 binds RBD with high affinity but does not perturb ACE2 binding. (**A**) FSEC of RBD in the absence (black) and presence (blue) of SR31. (**B**) Bio-layer interferometry (BLI) assay with RBD immobilized and SR31 as analyte at three concentrations (nM). (**C**) Analytic fluorescence-activated cell sorting (FACS) of HEK293T cells expressing the full-length S without staining (red), or with staining in the presence of PBS (***i***) or indicated sybodies (***ii-iv***) (cyan). (**D**) Pull-down of S expressed on the surface of SARS-CoV-2 pseudoviruses using His-tagged sybodies immobilized on Ni-NTA resin. Lanes 1–4 show the elution and lanes 5–8 show the input. Immunoblot bands of the full-length S and the S1 subunit were detected using anti-S1 antibody and conjugated secondary antibodies. Characteristically [4], the full-length S of SARS-CoV-2 is mostly processed. Images of the blot with different exposure are shown (***i*, *ii***). The bright-field image of the prestained molecular marker was merged with the chemiluminescent image of the immunoblots. (**E**) SR31 does not inhibit ACE2 binding. An RBD-coated sensor saturated with SR31 was soaked in 50 nM of SR31 with (blue) and without (black) 25 nM ACE2. As a control, the assay was performed with RBD immobilized and ACE2 as analyte (red). (**F**) Purification (SEC and SDS-PAGE) and crystallization of the RBD-SR31 complex. Void volume (V_o_) and total volume (V_t_) are appropriately labeled.

One such sybodies, named SR31, was characterized in this study. In analytic fluorescence-detection size exclusion chromatography (FSEC), RBD eluted earlier in the presence of SR31 compared to RBD alone (**Fig 1A**), suggesting the formation of a complex. Bio-layer interferometry analysis (**Fig 1B**) with RBD immobilized and SR31 as the analyte showed a *K*_D_ of 5.6 nM and an off-rate of 1 × 10^−3^ s^-1^. SR31, but not an irrelevant sybody (Sb66) that targets a green fluorescent protein [41], could bind to S expressed on the surface of HEK293T cells based on a cell surface staining assay measured by flow cytometry (**Fig 1C**). In addition, SR31, but not Sb66, could pull-down S protein expressed on the surface of SARS-CoV-2 pseudoviruses (**Fig 1D**). As expected, the previously identified [35] neutralizing MR6 sybody can also recognize S expressed at the cell surface or onto pseudovirus (**Fig 1C and 1D**). The results raise the possibility that SR31 binds S-RBD without impairing its receptor-binding capacity. Indeed, competitive bio-layer interferometry analysis showed nearly identical binding profiles of RBD-ACE2 in the absence or presence of SR31 (**Fig 1E**).

### Structure of SR31 in complex with RBD

To characterize the SR31-RBD interactions in detail, we purified the complex (**Fig 1F**) and obtained crystals (**Fig 1F**) that diffracted to 1.97 Å resolution (**Table 1**). The structure was solved by molecular replacement using the published RBD and sybody structures (PDB IDs 6M0J and 5M13) [6, 42] as search models. The structure was refined to *R*_work_/*R*_free_ of 0.182/0.207 (**Table 1**). The asymmetric unit contained one molecule each for the RBD and SR31, indicating an expected 1:1 stoichiometry.

**Table 1:** Data collection and refinement statistics.

	SR31 + RBD	MR17-SR31 + RBD
**Data collection**		
Space group	P 3_1_ 2 1	P 6_5_ 2 2
Cell dimensions		
*a*, *b*, *c* (Å)	92.39, 92.39, 101.15	73.38, 73.38, 478.36
*α*, *β*, *γ* (°)	90, 90, 120	90, 90, 120
Wavelength (Å)	0.97854	0.98754
Resolution (Å)	19.61–1.97 (2.04–1.97)^*a*^	49.70–2.10 (2.16–2.10)
*R*_merge_	0.091 (1.425)	0.140 (1.409)
*R*_pim_	0.209 (0.336)	0.034 (0.373)
*I*/σ*I*	19.5 (1.7)	12.6 (2.0)
Completeness (%)	99.9 (100.0)	99.7 (96.6)
Multiplicity	19.8 (18.8)	18.6 (14.5)
CC* ^*b*^	1.000 (0.949)	0.999 (0.965)
**Refinement**		
Resolution (Å)	19.61–1.97	49.70–2.10
No. reflections	35,702	46,078
*R*_work_ / *R*_free_	0.1822 / 0.2071	0.1949 / 0.2359
No. atoms	2,916	3,892
Protein	2,592	3,437
Ligands	158	235
Solvent	166	220
No. residues	329	435
B-factors (Å^2^)	49.49	50.52
Protein	48.01	48.17
Ligand/ion	73.11	78.91
Solvent	50.19	56.78
R.m.s deviations		
Bond lengths (Å)	0.008	0.008
Bond angles (°)	0.870	0.830
Ramachandran		
Favoured (%)	96.62	97.18
Allowed (%)	3.38	2.82
Outlier (%)	0	0
**PDB ID**	7D2Z	7D30

*a* Highest resolution shell is shown in parenthesis.

b CC* = $\sqrt{\frac{{2CC}_{1/2}}{1+{CC}_{1/2}}}$

**Fig 2:**
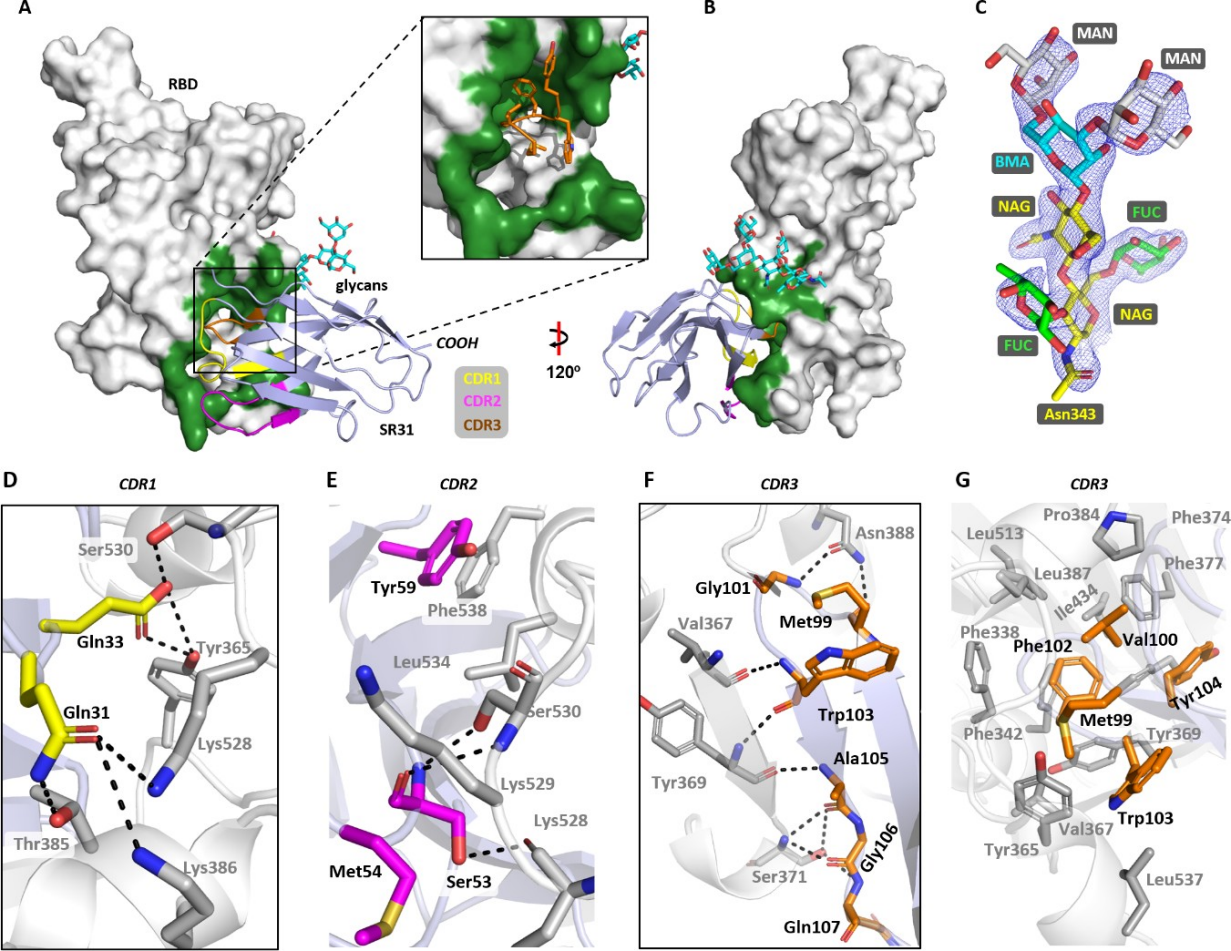
Crystal structure of the SR31-RBD complex. (**A**) The overall structure of SR31 (light blue) in complex with RBD (grey) which contains Asn343-linked glycans (cyan). The expanded view highlights a deep hydrophobic pocket (green) for CDR3 binding. (**B**) The overall structure viewed at a different angle. (**C**) 2*Fo-Fc* map of the Asn343-linked glycans. MAN, mannose; BMA, β-_D_-mannose; FUC, fucose; NAG, *N*-acetylglucosamine. (**D-G**) Detailed interactions between RBD and the CDR1 (**D**), CDR2 (**E**), and CDR3 (**F, G**). The hydrophobic network formed between CDR3 (orange) and the hydrophobic pocket in RBD (grey) is shown in **G**. Residues from SR31 are labeled with black texts and residues from RBD are labeled with grey texts. Dash lines indicate hydrogen bonds or salt bridges within 3.6 Å.

SR31 binds to the RBD sideways at a buried surface area of 1,386.3 Å^2^ (**Fig 2A**) (CDR1, 204.5 Å^2^; CDR2, 226.8 Å^2^, CDR3, 519.4 Å^2^; non-CDR, 710.3 Å^2^) which is significantly larger than that for the previously reported sybodies SR4 (727.4 Å^2^) and MR17 (853.9 Å^2^) [35]. The binding surface is near a heavily decorated glycosylation site, Asn343 (**Fig 2A, 2B and 2C**), which, although at an apparent strategic position to possibly divide the accessible surfaces for immune surveillance, does not show clashes with SR31. All three CDRs participated in the interaction by providing five (CDR1), three (CDR2), and nine H-bonds (CDR3) (**Fig 2D, 2E and 2F**). Peculiarly, the CDR3, which contains a cluster of hydrophobic side chains that include Met99, Val100, Phe102, Trp103, and Tyr104, is inserted into a greasy pocket (**Fig 2A**) in the RBD that is lined with twelve hydrophobic/aromatic residues (**Fig 2G**). Unlike salt bridges, hydrophobic interactions are more tolerant to environmental changes such as changes in pH and ionic strength. In addition, they are less specific and thus less likely to be affected by mutations. This binding mode thus makes SR31 an attractive candidate for detection purposes.

**Fig 3:**
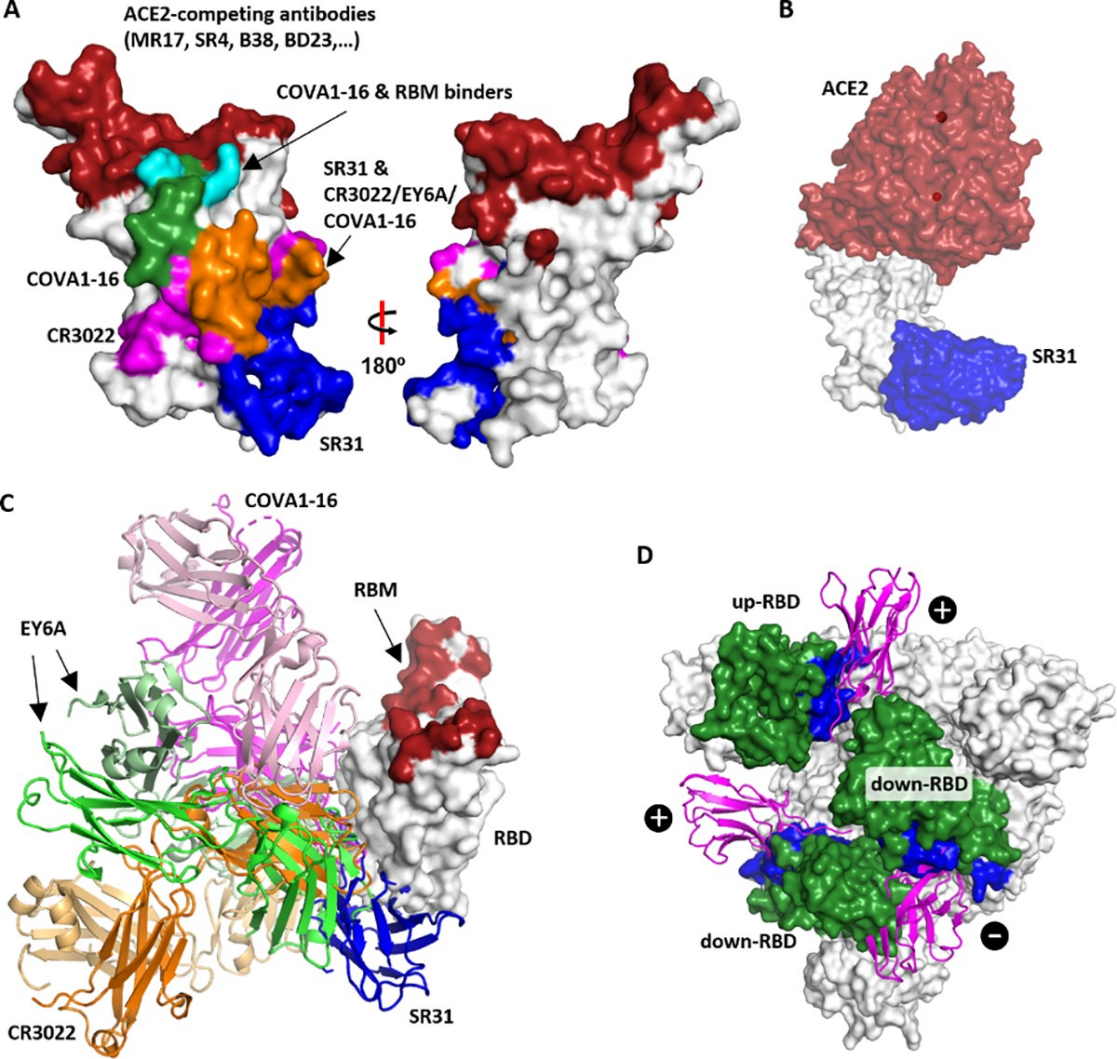
SR31 engages RBD at a site distal to the receptor-binding motif. (**A**) Comparison of the SR31 epitope with epitopes for other RBD-targeting nanobodies [22, 35, 36, 39] and mAbs [13–15, 19, 20, 23, 24, 26–28]. Red, the collective epitope of RBM-binders; blue, the SR31 epitope; magenta, the collective epitope of CR3022 and EY6A; green, the COVA1-16 epitope; cyan, the overlap between the epitopes of the RBM binders and COVA1-16; orange, the overlap between the CR3022/EY6A/COVA1-16 and SR31 epitope. (**B**) SR31 (blue) binds to RBD (grey) at a surface distal to the binding site of ACE2 (red). (**C**) Comparison of the binding mode between SR31 (blue) and three mAbs including CR3022 (orange and wheat), EY6A (green and pale green), and COVA1-16 (pink and magenta). RBD is shown as white surface with RBM highlighted in red. (**D**) The binding site of SR31 in the context of the S trimer at its pre-fusion ‘open’ state with one RBD in the ‘up’ conformation and two in the ‘down’ conformation. The structure (PDB ID 6yvb) [3] is viewed from the ‘top’ (perpendicular to the viral membrane). The SR31 epitope is shown in blue. The three RBDs are colored green. SR31 (magenta cartoon) is aligned to the S trimer (surface presentation) by superposing the SR31-RBD structure to each of the RBD. ‘+’, no or minor clashes; ‘-’, with severe clashes.

Apart from a few exceptions that neutralize SARS-CoV-2 by steric hindrance for ACE2-binding [34, 43] or by destructing S [12, 22], most structurally characterized RBD-targeting antibodies (human monoclonal antibodies and nanobodies) [8, 13–15, 19, 20, 22–24, 26–28, 34, 35, 37] engage the RBD at the receptor-binding motif (RBM) (**Fig 3A**), thus competing off ACE2 and preventing viral entry. Aligning the ACE2 structure to the SR31-RBD structure showed that the SR31-binding epitope is distant from the RBM (**Fig 3B**). Comparing the epitopes of existing monoclonal antibodies showed that the SR31 epitope partly overlaps with CR3022 [12], and the recently identified EY6A [22] and COVA1-16 [43] (**Fig 3A and 3C**). It has been established that the binding of the bulky CR3022/EY6A at the interface between RBD and the N-terminal domain (NTD) of the adjacent monomer destabilizes the S trimer and converts the pre-fusion conformation to the infection-incompetent post-fusion state, thus conferring neutralization activity [21, 22]. Despite the epitope overlapping, SR31 approaches RBD at a different angle to that of CR3022 (**Fig 3C**). This angular difference, together with its minute size, may allow SR31 to bind two of the three sites in the ‘open’-S [3]: the ‘up-RBD’ and the ‘down-RBD’ at the clockwise monomer (**Fig 3D**) without destructing S. Similarly, despite epitope overlap (**Fig 3A**), the binding of SR31 does not cause steric hindrance for ACE2-binding (**Fig 3B**) as is the case for COVA1-16 (**Fig 3C**) [43]. Taken together, the structural data rationalize the high-affinity binding between SR31 and RBD, and its inability to neutralize SARS-CoV-2.

### SR31-RBD structure suggests high RBD domain stability

**Fig 4:**
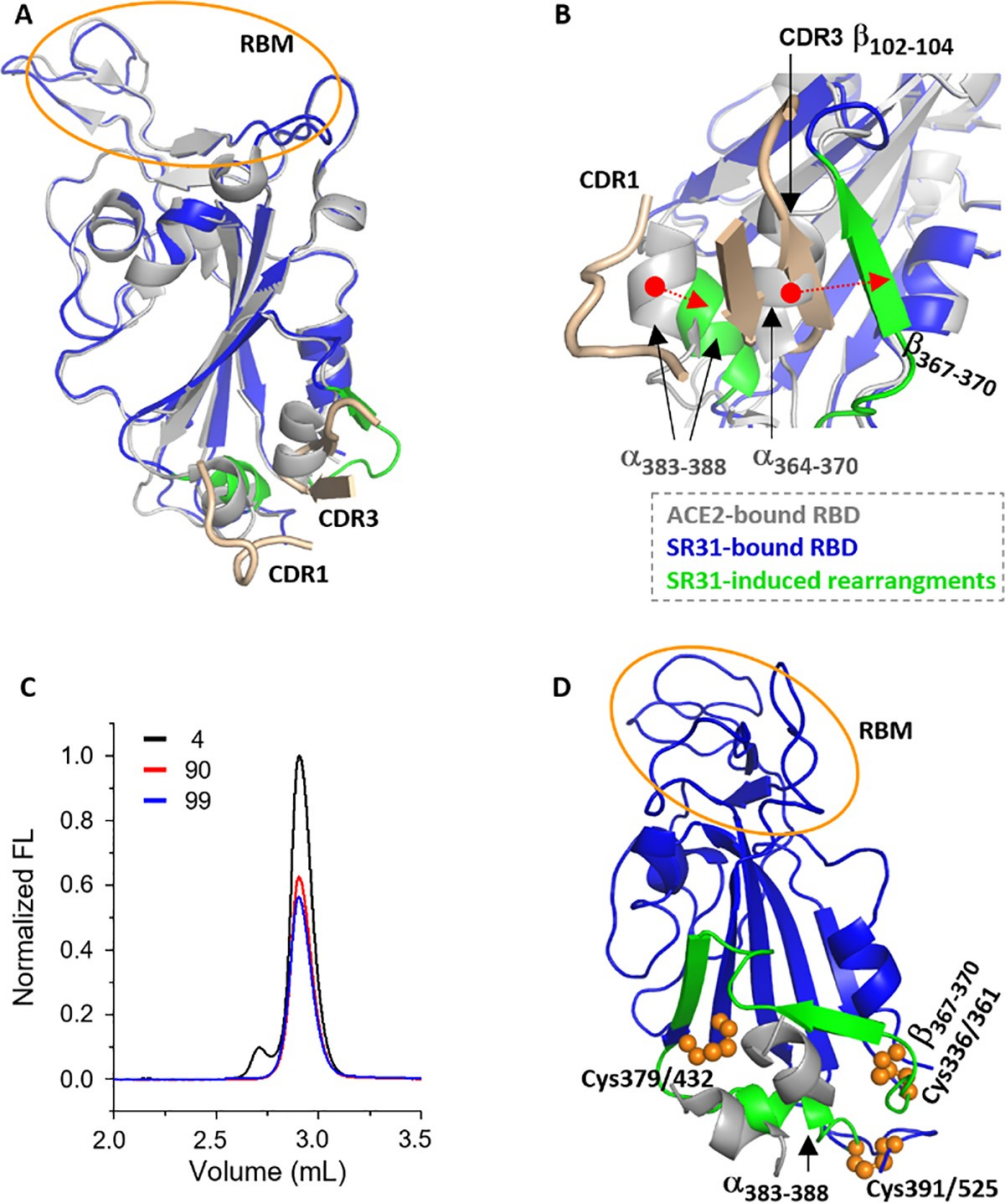
SR31 causes dramatic structural arrangements of RBD at the binding site without distorting the receptor-binding motif. (**A, B**) The overview (**A**) and expanded view (**B**) of the comparison between the ACE2-bound RBD (grey) and SR31-bound RBD (blue). SR31-binding deforms the RBD at the binding site (green) but not at the RBM region (yellow circle). The two SR31 CDRs involved in the deformation are colored wheat. In **B**, two structural rearrangements (green) are shown at a different angle. The α_383–388_ helix in the ACE2-bound form is pushed towards the RBD core, and the short helix α_364–370_ is transformed into a β-strand (β_367–370_) which forms a parallel β-sheet with β_102–104_ from SR31 CDR3. (**C**) An indirect stability assay of the RBD using fluorescence-detection size exclusion chromatography. The RBD was incubated at 4°C (black), 90°C (red), and 99°C (blue) for 20 min before loaded onto an analytical column for gel filtration. The retention profile of RBD was monitored by intrinsic tryptophan fluorescence. The void volume is 1.9 mL and the total volume is 4.5 mL. The chromatogram beyond 3.5 mL (not displayed) only showed background-level fluorescence. (**D**) Three disulfide bonds (orange spheres) segregate the two motifs (α_383–388_ and β_367–370_, green) from the RBM (orange cycle). α_383–388_ is tethered between Cys379/432 and Cys391/525; β_367–360_ is tethered between Cys379/432 and Cys336/361.

Structure alignment of SR31-RBD with ACE2-RBD revealed that the two RBD structures were overall very similar with a Cα RMSD of 0.45 Å (**Fig 4A**). Nevertheless, significant structural rearrangements at the binding interface were observed (**Fig 4A and 4B**). Specifically, the small α-helix α_364–370_ (numbers mark residues from start to end) moves towards the direction of RBM by a dramatic distance of ~8.0 Å and transforms to a short β-sheet (β_367–370_) which in turn forms a parallel β-sheet pair with β_102–104_ in the CDR3 region. In addition, nudged by the CDR1, the short helix α_383–388_ swings towards the RBD core by ~4.0 Å.

Remarkably, the dramatic rearrangements did not cause appreciable conformational changes of RBM (**Fig 4A**), nor did it affect ACE2 binding (**Fig 1E**). Given that RBD is a relatively small entity, and that the two surfaces are relatively close (~25 Å), this was somewhat unexpected. A probable explanation is that the core region of RBD is relatively rigid so that the RBM surface is not easily distorted allosterically. Because rigidity often correlates with heat stability [44], we measured the apparent melting temperature (*T*_m_) of RBD using a fluorescence-detection size exclusion chromatography thermostability assay [45]. Compared with glycoproteins with a similar size (erythropoietin, 21.3 kDa, *T*_m_ of 56°C with 10-min heating; human granulocyte colony-stimulating factor, 21.2 kDa, *T*_m_ of 62.5°C at a ramping rate of 1°C min^-1^; interferon beta, 22.3 kDa, *T*_m_ of ~70°C at a ramping rate of 2°C min^-1^) [46–48], RBD (22.6 kDa) showed much higher heat stability, with an apparent *T*_m_ of greater than 95°C (20-min heating) (**Fig 4C**).

Intriguingly, the rearrangement happens in a region that is rich in disulfide bonds. Specifically, β_367–370_ is tethered between the disulfide pairs Cys379-Cys432 and Cys336-Cys361, and α_383–388_ bridges Cys379-Cys432 and Cys-391-Cys525 (**Fig 4D**). Thus, the three disulfide bonds segregate the two local motifs from the rest of RBD, preventing these conformational changes from propagating through the domain.

### SR31 is a non-competing sybody for RBM binders

**Fig 5:**
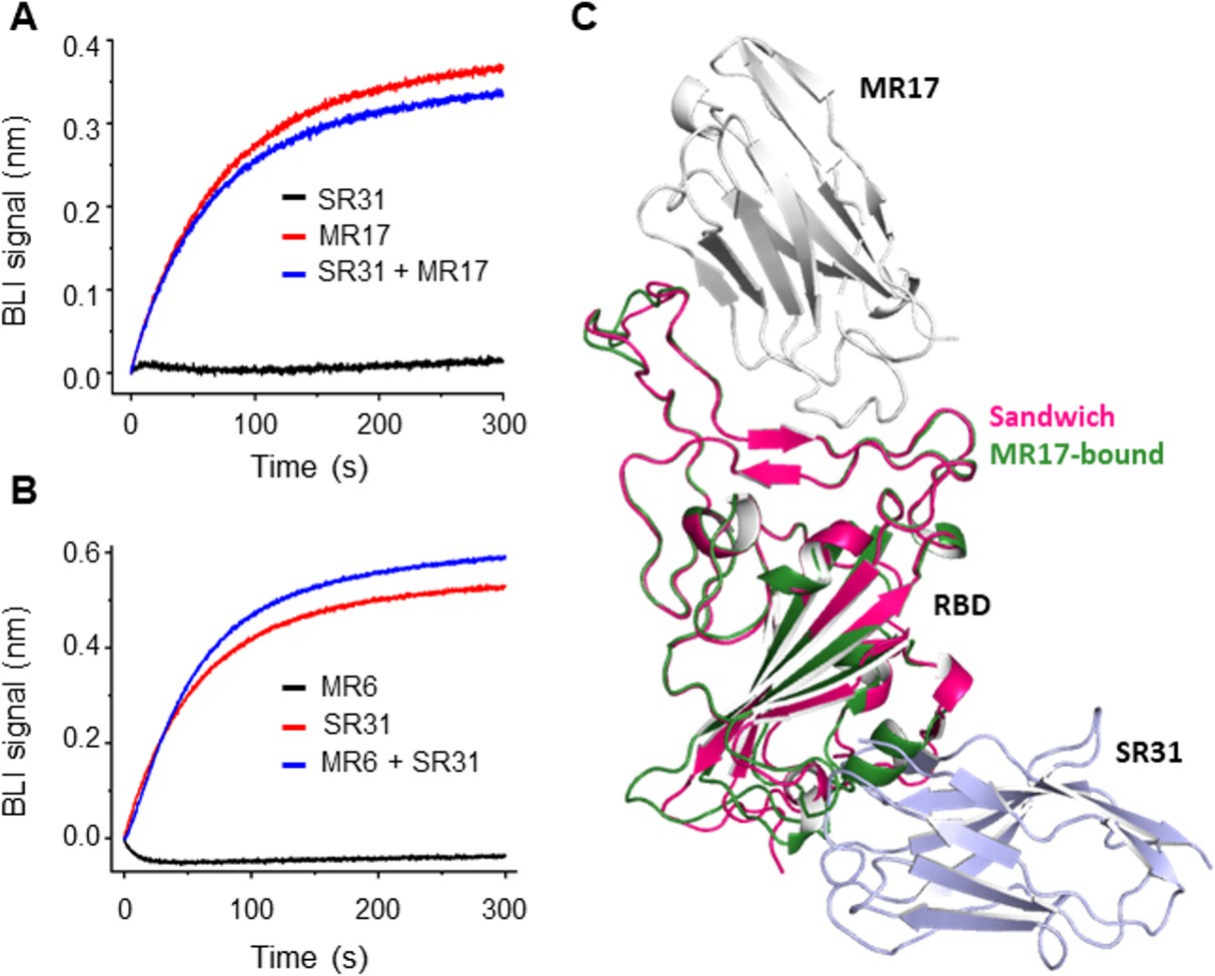
SR31 could pair with RBM nanobodies to bind RBD. (**A, B**) SR31 does not interfere with MR17 (**A**) or MR6 (**B**) for RBD-binding. In **A**, an RBD-coated sensor was pre-saturated in 200 nM of SR31 before incubating with SR31 alone (black) or a mixture (blue) of SR31 and MR17/MR6. In **B**, the sensor was saturated with MR6 before analyzed with SR31. For control purposes, the binding between RBD and the sybody used in the pre-incubation was also characterized (red). (**C**) Alignment of the biparatopic sybody (MR17-SR31)-RBD structure (MR17, grey; SR31, light blue; RBD, red) with the MR17-RBD structure (RBD, green) (PDB ID 7c8w) [35].

The neutral feature of SR31 so far suggests it could bind to RBD in addition to RBM binders such as MR17 and SR4 [35]. Indeed, BLI assays showed no competition between SR31 and MR17 (**Fig 5A**), indicating that RBD could bind both sybodies simultaneously. This non-competing feature was also observed in the case of MR6 (**Fig 5B**) which has been shown to neutralize SARS-CoV-2 pseudoviruses [35]. As further proof for the simultaneous binding, we constructed a biparatopic sybody by fusing SR31 to the C-terminal of MR17 via a Gly-Ser linker with 34 amino acids (**S1 Table**) and determined its structure in complex with RBD to 2.10 Å resolution (**Fig 5C**, and **Table 1**). This structure was similar to the individual MR17- and SR31-RBD complexes, with an overall Cα RMSD of 0.67 and 0.38 Å, respectively. Aligning the biparatopic sybody-RBD complex with the MR17-RBD structure revealed no appreciable changes at the MR17-binding surface (**Fig 5C**), reinforcing the idea that SR31-binding does not allosterically change the RBM surface and that SR31 is highly likely to be compatible with RBM-binding antibodies.

### SR31 fusion increases affinity and neutralization activity of MR17 and MR6

**Fig 6:**
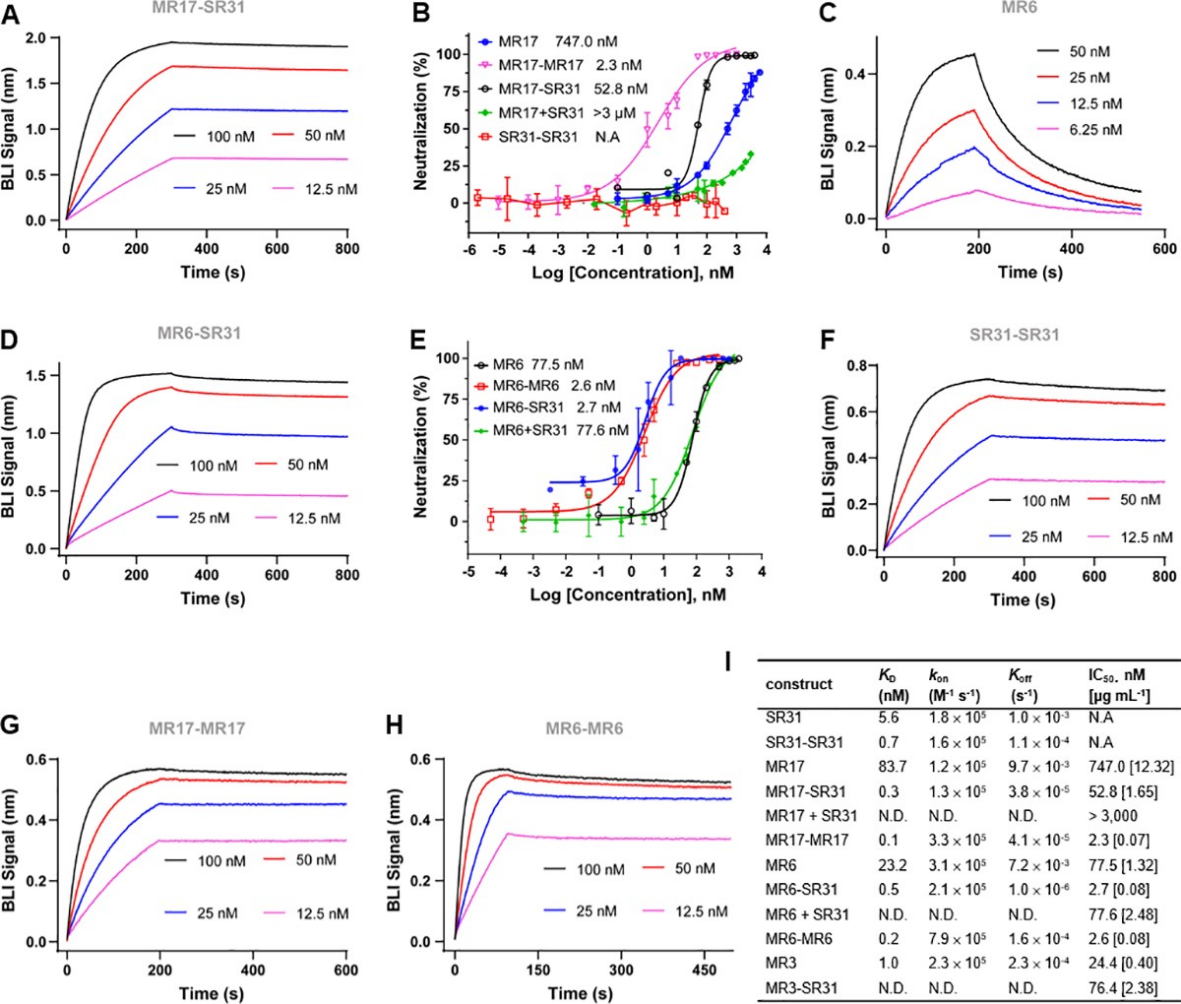
SR31 increases binding affinity and neutralization activity of two fusion partners. (**A**) BLI binding assay with immobilized RBD and the biparatopic sybody MR17-SR31 as analyte at increasing concentrations (nM). (**B**) Neutralization assay of MR17 (blue), MR17-MR17 (magenta), MR17-SR31 (black), the equimolar mix of MR17 and SR31 (MR17+SR31, green), and SR31-SR31 (red). (**C, D**) Binding kinetics for the RBD-binding by MR6 (**C**) or by MR6-SR31 (**D**). (**E**) Neutralization assay of MR6 (black), MR6-MR6 (red), MR6-SR31 (blue), and the equimolar mix of MR6 and SR31 (MR6+SR31, green). (**F, G, H**) BLI binding assay with immobilized RBD and the monoparatopic divalent sybody SR31-SR31 (**F**), MR17-MR17 (**G**), or MR6-MR6 (**H**) as analyte at increasing concentrations (nM). (**I**) Summary of the comparison between monovalent sybodies, SR31-fusion (biparatopic) sybodies, homo-fusion (monoparatopic) sybodies for binding kinetics and neutralization activities. Binding kinetics and neutralization data for MR17 and MR3 are from reference [35]. N.D., not determined; N.A., not applicable. In **B** and **E**, data are mean ± standard deviation from three independent experiments, and the x-axis indicates the concentration of the individual sybodies as opposed to the total sybody concentration.

Although SR31 does not neutralize SARS-CoV-2 pseudovirus itself, its high-affinity may help increase the affinity of other neutralizing nanobodies through avidity effects by fusion. Indeed, the biparatopic fusion MR17-SR31 (**S1 Table**) displayed a remarkable increase in binding affinity compared to SR31 or MR17 alone. Its *K*_D_ of 0.3 nM (**Fig 6A**) was lower than MR17 (*K*_D_ = 83.7 nM) [35] by 230 folds and lower than SR31 (*K*_D_ = 5.6 nM) by 17 folds. Consistently, MR17-SR31 neutralized SARS-CoV-2 pseudovirus 13 times more effectively (in molarity) than MR17 alone (**Fig 6B**). Interestingly, the IC_50_ of MR17 decreased in the presence of SR31 at equimolar concentrations (MR17 + SR31, **Fig 6B**). This antagonistic effect will be discussed.

That SR31 can enhance the potency of its fusion partner was also demonstrated in the case of MR6. In its free form, MR6 bound to RBD with a *K*_D_ of 23.2 nM (**Fig 6C**) and showed modest neutralizing activity with an IC_50_ of 1.32 μg mL^-1^ (77.5 nM). Fusing it to the N-terminal of SR31 in the same way as for MR17-SR31 (**S1 Table**) increased its affinity by over 40 folds, displaying a *K*_D_ of 0.5 nM (**Fig 6D**). In line with this, MR6-SR31 showed a 27-fold higher neutralization activity compared to MR6, with an IC_50_ of 2.7 nM (0.08 μg mL^-1^) (**Fig 6E**)_._ As a control, neutralization assay using equimolar mix of SR31 and MR6 yielded the same IC_50_ as that for MR6 (**Fig 6E**). Overall, the results confirm that the potency enhancement was mediated by SR31 fusion rather than its simple presence.

Interestingly, when fused to MR3, a neutralizing antibody that had a higher affinity (*K*_D_ = 1.0 nM) [35] than SR31, the neutralizing activity decreased by 2 folds (**Fig 6I**). Possible reasons include steric incompatibility caused by improper link length and allosteric effects. Future structural investigations to test these hypotheses directly are warranted.

To compare the binding affinity and the neutralization activity of SR31-fusion constructs with the monoparatopic divalent constructs, we additionally characterized three homo-fusion constructs: SR31-SR31, MR17-MR17, and MR6-MR6 (see **S1 Table** for sequence information). As expected, the divalent fusion increased binding affinity by 7 folds for SR31 (**Fig 6F and 6I**), over 800 folds for MR17 (**Fig 6G and 6I**), and by 115 folds for MR6 (**Fig 6H and 6I**); the improvement in affinity was expected based on our [35] and others’ previous experiences [22, 36, 37, 39, 49–53]. In neutralization assays, SR31-SR31 did not show any neutralizing activities (**Fig 6B**), as was the case for the monovalent form [35]. Consistent with the marked increase of affinity, the divalent monoparatopic fusion also drastically increased the neutralization activity, with a 324-fold increase for MR17-MR17 (**Fig 6B and 6I**), and a 29 fold for MR6-MR6 (**Fig 6E and 6I**). Interestingly, while the neutralization activity of MR6-SR31 was comparable to that of MR6-MR6 (**Fig 6E and 6I**), the neutralization potency of the MR17-MR17 was superior to the MR17-SR31 (**Fig 6B and 6I**). For reasons that will be discussed, the SR31-fusion does not necessarily provide more increase in potency than homo-fusion. Nevertheless, because the use of SR31 does not conflict with homo-fusion owing to the non-competing binding mode, SR31 may help further increase the potency of monoparatopic nanobodies that are already multivalent.

## Discussion

The spike protein of SARS-CoV-2 is highly immunogenic and has been studied extensively in the context of antibody discovery. Most of these antibodies target RBD [54, 55]. Among the many RBD-targeting neutralizing antibodies, only a handful have their epitopes accurately determined by structural characterizations which can be non-trivial. In the absence of structural data, techniques such as hydrogen-deuterium exchange mass spectrometry [23] and mutagenesis mapping [40] can be used to determine epitope, but these methods are also time-consuming. Alternatively, epitopes may be probed by competitive binding assays using antibodies with known epitopes [40]. Because nanobodies are relatively easy to produce, the availability of nanobodies that collectively recognize a wide spectrum of epitopes can be a useful toolkit in this regard. They may also be used to select binders with new epitopes by including them as pre-formed sybody-RBD complexes during *in vitro* selection (and thus excluding overlapping binders). SR31, together with other reported nanobodies [22, 34, 36, 37, 40, 49], presents a useful research tool in the abovementioned applications.

Because of the minute size, SR31 may be a versatile ‘add-on’ to existing monoclonal antibodies, scFv fragments, human VH domains, and other nanobodies [55] to enhance their affinity and potency, especially for those with modest neutralizing activities. Compared to other techniques such as random mutagenesis [22] and structure-based design [35], the fusion technique is more rapid and less involving. In addition, due to its small size and high stability, SR31 may be chemically modified as a vector to deliver small-molecule inhibitors to specifically target SARS-CoV-2.

This work generated two biparatopic sybodies, MR17-SR31 and MR6-SR31. Compared with monoparatopic divalent nanobodies or monoclonal antibodies, biparatopic nanobodies are more likely to be resistant to escape mutants because simultaneous mutations at two distinct and relatively remote epitopes should occur at a much lower rate than at a single epitope. Whether this is true for the biparatopic sybodies identified here remains to be tested. While the neutralizing activities of the biparatopic sybodies are comparable to some bivalent nanobodies and human VH domains in the literature [22, 36, 39, 51], we note the existence of a few ultra-potent nanobodies [50, 53], especially those with high valency. Because SR31 does not compete with MR17 or MR6, one could construct hexavalent sybodies with three copies each of SR31 and MR17/MR6 to further increase potency. SR31 may also be fused to ultra-potent nanobodies in the literature to make even tighter fusion nanobodies, and to increase their size for longer *in vivo* half-lives [29] which is an important characteristic for nanobody drugs.

Interestingly, SR31 decreased MR17’s potency when included at equimolar concentrations. While somewhat unexpected, this may reflect the complexity of the trimeric S protein. Thus, SR31-binding may cause slight conformational changes of S in a way that the MR17-binding surface is partially protected by adjacent structures such as the N-terminal domain from other subunits.

The reason for the superior neutralization activity of the homo-fusion constructs (MR17-MR17) over SR31-fusion constructs can be complex. First, one molecule of the homo-fusion sybody could potentially block two of the three RBMs when S assumes a ‘two up-RBD’ conformation [56]. By contrast, the biparatopic SR31-fusion constructs could only act on one RBM, leaving two free RBMs that could bind the dimeric ACE2 much more efficiently compared to S proteins with one accessible RBM. In this sense, the superior potency of homo-fusion constructs is not surprising. Second, the potency of fusion constructs depends on, to some degree, the linker type and length, and the optimal linker for different constructs may differ. These factors make it difficult to directly compare different divalent types in a meaningful way. Third, as suggested by the neutralization assay with equimolar mixes of SR31 and MR17, the presence of SR31 may affect the recognition of RBM by MR17 in the context of trimeric S, despite they bind to the isolated RBD in a non-competing manner (**Fig 5A and 5C**). Practically, because SR31 does not directly compete with MR17 or MR6 for epitope, it is not exclusive for RBM-binders regarding multivalent engineering. Rather, the gained potency by SR31 fusion on whatever existing form, if present, will be additive.

In summary, we have structurally characterized SR31, a high-affinity nanobody against SARS-CoV-2 RBD. Although lacking neutralizing activity alone, SR31 is an attractive biparatopic partner for RBM-binders owing to its distinct epitope from RBM. Our work presents a generally useful strategy and offers a simple and fast approach to enhance the potency of modestly active antibodies against SARS-CoV-2.

## Materials and methods

### Protein purification

SARS-CoV-2 RBD was expressed essentially as described [35]. Briefly, a DNA fragment encoding, from N- to C-terminus, residues 330–541 of SARS-CoV-2 S, a Gly-Thr linker, the 3C protease site (LEVLFQGP), a Gly-Ser linker, the Avi tag (GLNDIFEAQKIEWHE), a Ser-Gly linker, and a deca-His tag were cloned into the pFastBac-based vector. Baculovirus was generated in *Sf9* cells following the Invitrogen Bac-to-Bac transfection protocol. High Five insect cells were infected with P3 virus. Medium was collected 48–60 h post infection and incubated with 3.0 mL of Ni-Sepharose Excel resin (Cat 17-3712-03, GE Healthcare) pre-equilibrated with Buffer A (150 mM NaCl, 20 mM Tris pH8.0). After batch binding for 2–3 h, the resin was washed with 20 mM of imidazole in Buffer A and eluted with 300 mM imidazole in Buffer A.

C-terminally His-tagged sybodies were expressed in *Escherichia coli* MC1061 cells. Cells carrying sybody-encoding genes in the vector pSb-init [42, 57] were grown in Terrific Broth (TB, 0.17 M KH_2_PO_4_ and 0.72 M K_2_HPO_4_, 1.2%(w/v) tryptone, 2.4%(w/v) yeast extract, 0.4% (v/v) glycerol) plus 25 mg L^-1^ chloramphenicol to OD_600_ of 0.5 at 37°C. Cells were allowed to grow for another 1.5 h at 22°C before induced with 0.02%(w/v) arabinose for 17 h. Cells were harvested and lysed by osmotic shock as follows. Cell suspension in 20 mL of TES-high Buffer (0.5 M sucrose, 0.5 mM EDTA, and 0.2 M Tris-HCl pH 8.0) was first incubated at 4°C for 30 min for dehydration. To the cell suspension, 40 mL of ice-cold MilliQ H_2_O was added for rehydration at 4°C for 1 h. The suspension was centrifuged at 20,000×g at 4°C for 30 min to collect supernatant which contained periplasmic extracts. Appropriate buffers were added to the supernatant to have a final concentration of 150 mM NaCl, 2 mM MgCl_2,_ and 20 mM imidazole. The supernatant was then incubated with Ni-NTA resin pre-equilibrated with 20 mM of imidazole in Buffer B (150 mM NaCl and 20 mM Tris HCl pH 8.0). After batch-binding for 2 h, the Ni-NTA resin was subsequently washed and eluted with 30 mM and 300 mM imidazole in Buffer B, before desalted into PBS buffer.

Divalent sybodies were engineered to have SR31 at the N-terminal and other sybodies at the C-terminal. The DNA fragments were linked together with sequences encoding Gly-Ser linkers at specified length by Gibson Assembly and insertion PCR. Divalent sybodies were expressed and purified essentially as for the monovalent sybodies.

For crystallization, SR31 or MR17-SR31 was mixed with RBD at a 1:1.5 molar ratio. The mixture was then loaded onto a Superdex 200 column for gel filtration. Fractions containing the complex were pooled and concentrated to 10 mg mL^-1^.

### Fluorescence-detection size-exclusion chromatography (FSEC)

To screen RBD binders by size exclusion chromatography (SEC) using unpurified sybodies, RBD was fluorescently labeled as follows. First, the avi-tagged RBD was enzymatically biotinylated. It was then incubated with fluorescein-labeled streptavidin. The bright fluorescence of the RBD-streptavidin complex at visible wavelength enables convenient and specific monitoring of RBD SEC behavior without the need for purification.

To assess if sybody of interests binds RBD, purified or unpurified sybody was mixed with the fluorescent RBD before injecting on an analytical SEC column connected to an HPLC machine equipped with a fluorescence detector. The retention profile was then recorded by the fluorescence signal at the excitation/emission pair of 482/508 nm.

### Bio-layer interferometry assay

Bio-layer interferometry (BLI) was used to measure binding kinetics between sybodies and RBD. Biotinylated RBD (2 μg mL^-1^ in 0.005%(v/v) Tween 20, 150 mM NaCl, 20 mM Tris HCl pH 8.0) was first bound to the SA sensor (Cat 18–5019, ForteBio) which was coated with streptavidin. The sensor was equilibrated (baseline) for 120 s at 30°C. The sensor was then soaked with sybodies at various concentrations (association) for 100–300 s, before moving into a sybody-free buffer for dissociation. BLI signal was monitored during the whole process. Data were fitted with a 1:1 stoichiometry using the build-in software Analysis 10.0 for kinetic parameters. For competitive assay of the RBD between SR31 and ACE2, the RBD-coated sensor was saturated in 200 nM of SR31, before soaked in 25 nM SR31 with or without 25 nM of ACE2. As a control, BLI assays were also carried out by soaking the RBD-coated sensor in ACE2 without SR31. For competitive RBD-binding assays for different sybodies, the assays were carried out in the same manner as described above.

### Sybody immunoprecipitation

SARS-CoV-2 pseudotyped particles were produced as described below in neutralization assay. Sybodies (10 μL of a 30-μM stock) were incubated with 390 μL of pseudotyped particles for 4 h at room temperature (20–22°C) with gentle rotation. A fraction of the input mixture was mixed with 5× Laemmli loading buffer (10% SDS, 0.2% bromophenol blue, 50% glycerol, 1% β-mercaptoethanol, 250 mM Tris-HCl pH 6.8) and boiled for 5 min. Ni Sepharose 6 Fast Flow beads (40 μL, GE Healthcare, #17-5318-01) were incubated with the mixture at 4°C overnight with gentle rotation. The sample was washed 3 times with 1× binding buffer (0.5 M NaCl, 40 mM imidazole, 20 mM sodium phosphate pH 7.4) by resuspension and centrifugation at 5,000×g for 1 min, then eluted with 1× elution buffer (0.5 M NaCl, 0.5 M imidazole, 20 mM sodium phosphate pH 7.4) by boiling at 100°C for 10 minutes. Samples were mixed with Laemmli loading buffer before loaded on SDS-PAGE gels (EpiZyme, PG111) and analyzed by immunoblot using S1 of SARS-CoV-2 monoclonal mouse antibody (Sino Biological, 40591-MM42) and HRP-conjugated Goat-anti-mouse antibody (Proteintech, SA00001-1).

### Cell surface *s*taining assays using sybody

HEK293T cells were transfected with a plasmid DNA encoding SARS-CoV-2 spike protein once cells reached approximately 50–70% confluency using polyethylenimine. 24 hours post transfection, cells were rinsed with PBS then detached with EDTA. After cell counting, 5×10^5^ cells were washed with 1 mL PBFA (PBS supplemented with 2% fetal calf serum and 0.1% sodium azide) by resuspension and centrifugation at 800×g for 3 min at 4°C. The pellet was subsequently incubated in 100 μL primary antibody (sybodies at a final concentration of 3 μM or an equal volume of PBS; diluted in PBFA) at 4°C for 30 min. After washing in PBFA, cells were incubated in 100 μL of secondary antibody (1:100, Mouse anti-His-tag mAb, Abclonal, #AE003; then with Goat Anti-Mouse IgG(H+L)-FITC, SouthernBiotech, #1036–02; diluted in PBFA) at 4°C for 30 min and washed twice with 1 mL PBFA. 100,000 live cells in ice-cold PBS were analyzed by fluorescence-activated cell sorting (FACS). As a control group, the same amount of cells were also analyzed by FACS with no antibodies (sybodies and secondary antibodies).

### Pseudotyped particle production and neutralizing assay

To generate retroviral pseudotyped particles, HEK293T cells were co-transfected with the vectors expressing the various viral envelope glycoproteins, the murine leukemia virus core/packaging components (MLV Gag-Pol), and a retroviral vector expressing the green fluorescence protein (GFP). The S protein of SARS-CoV and SARS-CoV-2 in the phCMV plasmid were truncated by 19 amino acids at the C-terminus. Pseudotyped particles were harvested 48 h post-transfection by centrifugation and the supernatant was filtered through a 0.45-μm membrane before neutralization assays.

Fifty microliters of VeroE6-hACE2 cells (10^4^ cells/well) were seeded in a 48-well plate. After 24 h, cells were infected with 100 μL of pseudovirus. When sybodies were included, they were incubated with the pseudovirus for 1 h at 37°C before infection.

After 6 h of co-incubation, the supernatants were removed and the cells were incubated in medium (Dulbecco’s modified Eagle’s medium-2% fetal calf serum) for 72 h at 37°C. GFP expression was determined by fluorescence-activated flow cytometry analysis. Cells incubated with medium-only were used as a control to calculate percent inhibition.

### Crystallization

Crystallization experiments were conducted using a Gryphon LCP robot. A two-well sitting-drop plate was filled with 70 μL of the precipitant solution as the reservoir. To each well, 100 nL of protein solution was touch-dispensed using the LCP dispenser of the robot. The protein solution was then mixed with 100 nL of precipitant solution delivered by the 96-headed tips. Plates were sealed with transparent tape (Cat HR4-506, Hampton research) and incubated in a Rocker Imager 1000 at 20°C for automated imaging.

Crystals for the SR31-RBD complex were grown in 2.0 M Sodium formate, 0.1 M Sodium acetate trihydrate pH 4.6. Cryo protection was achieved by adding 20%(v/v) glycerol to the mother liquor condition. Crystals for the MR17-SR31-RBD complex were grown in 0.1 M cadmium chloride, 0.1 M Na-acetate pH 4.5, 30% PEG 400, 4% v/v (±)-1,3-butanediol. Cryo protection was achieved by adding 20%(v/v) glycerol in the mother liquor condition.

Desired crystals were cryo-protected, harvested using a MiTeGen loop under a microscope, and flash-cooled in liquid nitrogen before diffraction.

### Data collection and structure determination

X-ray diffraction data were collected at beamline BL19U1 [58] at Shanghai Synchrotron Radiation Facility with a 50 × 50 μm beam on a Pilatus 6M detector, with oscillation of 0.5° and a wavelength of 0.97853 Å. Data were integrated using the software XDS [59], and scaled and merged using Aimless [60]. The SR31-RBD structure was solved by molecular replacement using Phaser [61] with PDB IDs 6M0J and 5M13 [42] as the search model. The MR17-SR31-RBD structure was solved using the SR31-RBD and MR17 structure [35] as search models. The models were manually adjusted as guided by the 2F_o_-F_c_ maps in Coot [62], and refined using Phenix [63]. Structures were visualized using PyMol [64].

## Supporting information

S1 Table Sequences of biparatopic sybodies. (DOCX)
