# Supplementary material for: A high-affinity RBD-targeting nanobody improves fusion partner’s potency against SARS-CoV-2
Source: PLoS Pathog. 2021 Mar 3;17(3):e1009328. doi: 10.1371/journal.ppat.1009328 (PMC7959386; doi:10.1371/journal.ppat.1009328)
Supplement: S1 Table — (DOCX) [file ppat.1009328.s001.docx]

**S1 Table. Sequences of biparatopic sybodies.**

| **Sybody** | **Sequence*^a^*** |
| --- | --- |
| SR31-SR31 | QVQLVESGGGLVQAGGSLRLSCAASGFPVWQGEMAWYRQAPGKEREWVAAISSMGYKTYYADSVKGRFTISRDNAKNTVYLQMNSLKPEDTAVYYCAVMVGFWYAGQGTQVTVS*GGGGSGGGGSGGGGSGGGGSGGGGSGGGGSGSSS*QVQLVESGGGLVQAGGSLRLSCAASGFPVGRASMWWYRQAPGKEREWVAAISSYGWVTAYADSVKGRFTISRDNAKNTVYLQMNSLKPEDTAVYYCEVSVGTGYRGQGTQVTVS |
| MR17-SR31 | QVQLVESGGGLVQAGGSLRLSCAASGFPVEVWRMEWYRQAPGKEREGVAAIESYGHGTRYADSVKGRFTISRDNAKNTVYLQMNSLKPEDTAVYYCNVKDDGQLAYHYDYWGQGTQVTVS*GGGGSGGGGSGGGGSGGGGSGGGGSGGGGSGSSS*QVQLVESGGGLVQAGGSLRLSCAASGFPVWQGEMAWYRQAPGKEREWVAAISSMGYKTYYADSVKGRFTISRDNAKNTVYLQMNSLKPEDTAVYYCAVMVGFWYAGQGTQVTVS |
| MR17-MR17 | QVQLVESGGGLVQAGGSLRLSCAASGFPVEVWRMEWYRQAPGKEREGVAAIESYGHGTRYADSVKGRFTISRDNAKNTVYLQMNSLKPEDTAVYYCNVKDDGQLAYHYDYWGQGTQVTVS*GGGGSGGGGSGGGGSGGGGSGGGGSGGGGSGSSS*QVQLVESGGGLVQAGGSLRLSCAASGFPVEVWRMEWYRQAPGKEREGVAAIESYGHGTRYADSVKGRFTISRDNAKNTVYLQMNSLKPEDTAVYYCNVKDDGQLAYHYDYWGQGTQVTVS |
| MR6-SR31 | QVQLVESGGGLVQAGGSLRLSCAASGFPVEDTWMEWYRQAPGKEREWVAAITSWGFKTYYADSVKGRFTISRDNAKNTVYLQMNSLKPEDTAVYYCNVKDEGDTSASYDYWGQGTQVTVS*GGGGSGGGGSGGGGSGSSS*QVQLVESGGGLVQAGGSLRLSCAASGFPVWQGEMAWYRQAPGKEREWVAAISSMGYKTYYADSVKGRFTISRDNAKNTVYLQMNSLKPEDTAVYYCAVMVGFWYAGQGTQVTVS |
| MR6-MR6 | QVQLVESGGGLVQAGGSLRLSCAASGFPVEDTWMEWYRQAPGKEREWVAAITSWGFKTYYADSVKGRFTISRDNAKNTVYLQMNSLKPEDTAVYYCNVKDEGDTSASYDYWGQGTQVTVS*GGGGSGGGGSGGGGSGGGGSGGGGSGGGGSGSSS*QVQLVESGGGLVQAGGSLRLSCAASGFPVEDTWMEWYRQAPGKEREWVAAITSWGFKTYYADSVKGRFTISRDNAKNTVYLQMNSLKPEDTAVYYCNVKDEGDTSASYDYWGQGTQVTVS |
| SR31-MR3 | QVQLVESGGGLVQAGGSLRLSCAASGFPVNAHFMYWYRQAPGKEREWVAAIYSYGRTLYADSVKGRFTISRDNAKNTVYLQMNSLKPEDTAVYYCNVKDYGAASWEYDYWGQGTQVTVS*GGGGSGGGGSGGGGSGGGGSGGGGSGGGGSGSSS*QVQLVESGGGLVQAGGSLRLSCAASGFPVWQGEMAWYRQAPGKEREWVAAISSMGYKTYYADSVKGRFTISRDNAKNTVYLQMNSLKPEDTAVYYCAVMVGFWYAGQGTQVTVS |

*^a^*The sequences include ‘GSSS’ at the N-terminal and ‘AGRAGEQKLISEEDLNSAVDHHHHHH’ at the C-terminal which contains a myc-tag (underlined) for ELISA and a hexahistidine tag for purification.
