# Supplementary material for: A high-affinity RBD-targeting nanobody improves fusion partner’s potency against SARS-CoV-2
Source: PLoS Pathog. 2021 Mar 3;17(3):e1009328. doi: 10.1371/journal.ppat.1009328 (PMC7959386; doi:10.1371/journal.ppat.1009328)
Supplement: Attachment — Submitted filename: Response_letter_DL_20201218.pdf [file ppat.1009328.s002.pdf]

Reviewer #1:

The authors report the isolation and characterization of nanobody SR31 derived from a synthetic library by ribosome display using SARS-CoV-2 RBD as the target. SR31 can bind to SARS-CoV-2 RBD, does not compete for hACE2 binding and does not neutralize SARS-CoV-2. The co-crystal structure of SR31 with RBD was determined, which revealed a hydrophobic interface and a large epitope. SR31 and RBD undergo an induced fit upon binding. The structures of SR32 fused to nanobody MR17 and MR6, in complex with the RBD were also determined. The pseudotype virus neutralizing activity of MR17 and MR6 increased 27-40 fold when merged with SR31. Complete neutralization was achieved with MR6 but not with SR31-MR6.

This is an interesting, in general well performed study.

We thank the reviewer for the supportive comments for our work.

Major remarks:

1. The authors should show that SR31 can bind to full length spike, either as a purified recombinant protein or when expressed on the surface of transfected cells. Such an experiment can demonstrate that the “greasy” epitope is accessible in full length spike.

We performed this critical experiment and the results showed that SR31 could bind to S expressed on the cell surface of HEK293T cells, and on the surface of SARS-CoV-2 pseudoviruses. Relevant text and figure information are below:

“SR31, but not an irrelevant sybody (Sb66) that targets a green fluorescent protein (*41*), could bind to S expressed on the surface of HEK293T cells based on a cell surface staining assay measured by flow cytometry (**Fig. 1C**). In addition, SR31, but not Sb66, could pull-down S protein expressed on the surface of SARS-CoV-2 pseudoviruses (**Fig. 1D**). As expected, the previously identified (*35*) neutralizing MR6 sybody can also recognize S expressed at the cell surface or onto pseudovirus (**Fig. 1C, 1D**).”

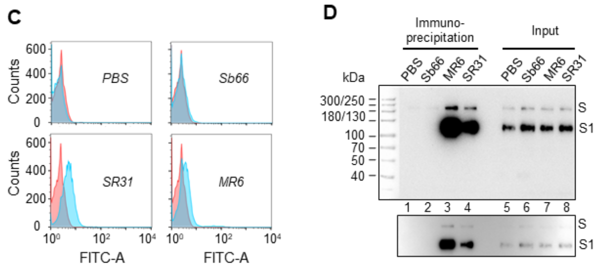

Fig. 1. SR31 binds RBD with high affinity but does not perturb ACE2 binding. (**C**) Analytic fluorescence-activated cell sorting (FACS) of HEK293 cells expressing the full-length S without staining (red), or with staining in the presence of PBS (***i***) or indicated sybodies (***ii-iv***) (cyan). (**D**) Pull-down of S expressed on the surface of SARS-CoV-2 pseudoviruses using His-

tagged sybodies immobilized on Ni-NTA resin. Lanes 1-4 show the elution and lanes 5-8 show the input. Immunoblot bands of the full-length S and the S1 subunit were detected using anti-S1 antibody and conjugated secondary antibodies. Characteristically (*43*), the full-length S of SARS-CoV-2 is mostly processed. Images of the blot with different exposure are shown (***i, ii***). The bright-field image of the prestained molecular marker was merged with the chemiluminescent image of the immunoblots.

2. It is important to show that the enhanced neutralizing activity of SR31-MR17 and SR31-MR6 is also or not observed when the respective nanobodies are simply mixed at equimolar concentrations in the neutralization assays.

We performed this critical control experiment. When added as an equimolar mixture, SR31 had no effect on the neutralizing activity of MR6 (Fig. 6E) but had a negative impact on the neutralizing activity of MR17 (Fig. 6B).

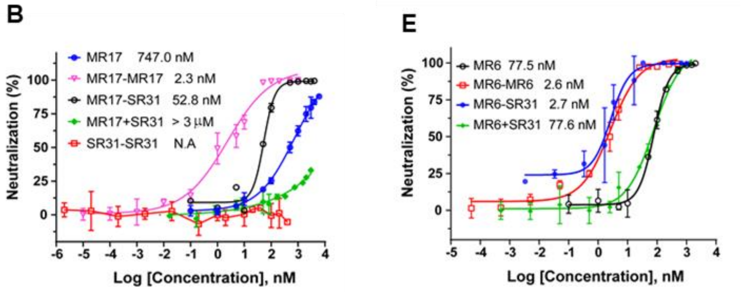

Fig. 6. SR31 increases binding affinity and neutralization activity of two fusion partners. (**B**) Neutralization assay of MR17 (blue), MR17-MR17 (magenta), MR17-SR31 (black), equimolar mix of MR17 and SR31 (MR17+SR31, green), and SR31-SR31 (red). (**E**) Neutralization assay of MR6 (black), MR6-MR6 (red), MR6-SR31 (blue), and equimolar mix of MR6 and SR31 (MR6+SR31, green). In **B** and **E**, data are mean ± standard deviation from three independent experiments, and the x-axis indicates concentration of the individual sybodies as opposed to the total sybody concentration.

The decreased neutralizing activity of MR17 upon addition of SR31 is discussed in the Discussion section:

"Interestingly, SR31 decreased MR17's potency when included at equimolar concentrations. While somewhat unexpected, this may reflect the complexity of the trimeric S protein. Thus, SR31-binding may cause slight conformational changes of S in a way that the MR17-binding surface is partially protected by adjacent structures such as the N-terminal domain from other subunits."

Other remarks:

1. Line 57: "nanobodies are known to cause little immunogenicity" is a too strong statement. There is only one nanobody-based biologic on the market for clinical use. Anti-drug antibodies against Alx-0171, a homotrivalent nanobody construct, have been reported in infants that were hospitalized due to RSV and were enrolled in phase 2 and 3 trials with this drug. "Nanobodies are considered to be poorly immunogenic in human" would be a more prudent statement.

Done. We thank the reviewer for the suggestion.

2. Line 88: most of the sybody binders should be "most of the RBD binders".

Done.

3. Line 93: "caused earlier retention" should be "eluted earlier".

Done.

4. Line 142: the nanobody against SARS-CoV-1 and -2 that is described in ref 34, does not bind to the RBM yet it competes with hACE2 and can neutralize SARS-CoV-2. Please correct the statement.

We thank the reviewer for catching this error.

The revised text now reads:

"Apart from a few exceptions that neutralize SARS-CoV-2 by steric hindrance for ACE2-binding (*34*, *43*) or by destroying S (*12*, *22*), most structurally characterized RBD-targeting antibodies (human monoclonal antibodies and nanobodies) (*8*, *13-15*, *19*, *20*, *22-24*, *26-28*, *34*, *35*, *37*) engage the RBD at the receptor-binding motif (RBM) (**Fig. 3A**), thus competing off ACE2 and preventing viral entry."

Reviewer #2:

In this manuscript, the authors report several crystal structures of nanobodies that bind to the receptor binding domain (RBD) or receptor binding motif (RBM) of SARS-CoV-2 spike. They have also characterized fusion constructs of synthetic nanobodies (sybodies) and found that biparatopic fusion of two nanobodies targeting different epitopes on the RBD increases their affinity and neutralization potency. While this work certainly sheds additional light on the already vast body of work on nanobodies in the context of SARS-CoV-2, there are several issues that need to be addressed before publication can be recommended.

We thank the reviewer sincerely for the constructive comments to improve our work.

Major comments

1. The main issue is the lack of many essential controls in the most important figure of the manuscript, Figure 6. For many SARS-CoV-2 antibodies and nanobodies, avidity effects have been described to contribute quite heavily to neutralization potency and binding (https://www.ncbi.nlm.nih.gov/pmc/articles/PMC7418720) Importantly, when describing the sybody fusion construct SR31-MR17, an essential comparison would be to compare it to SR31-SR31 and MR17-MR17 fusion constructs to be able to attribute the gain of affinity and neutralization capacity to the biparatopic nature of the construct and not simply because of increase of avidity. The same is true for Figure 6C-D-E-F. Especially since the predominant binding data is in the form of BLI and the density of the RBD on the biosensor will be quite high, it is very relevant and important to address bivalent monoparatopic constructs (SR31-SR31, MR17-MR17 etc.).

We performed these important control experiments and updated the manuscript:

"To compare the binding affinity and the neutralization activity of SR31-fusion constructs with the monoparatopic divalent constructs, we additionally characterized three homo-fusion constructs: SR31-SR31, MR17-MR17, and MR6-MR6 (see **Table S1** for sequence information). As expected, the divalent fusion increased binding affinity by 7 folds for SR31 (**Fig. 6F, 6I**), over 800 folds for MR17 (**Fig. 6G, 6I**), and by 115 folds for MR6 (**Fig. 6H, 6I**); the improvement in affinity was expected based on our (*35*) and others' previous experiences (*22, 36, 37, 39, 49*-*53*). In neutralization assays, SR31-SR31 did not show any neutralizing activities (**Fig. 6B**), as was the case for the monovalent form (*35*). Consistent with the marked increase of affinity, the divalent monoparatopic fusion also drastically increased the neutralization activity, with a 324-fold increase for MR17-MR17 (**Fig. 6B, 6I**), and a 29 fold for MR6-MR6 (**Fig. 6E, 6I**). Interestingly, while the neutralization activity of MR6-SR31 was comparable to that of MR6-MR6 (**Fig. 6E, 6I**), the neutralization potency of the MR17-MR17 was superior to the MR17-SR31 (**Fig. 6B, 6I**). For reasons that will be discussed, the SR31-fusion does not necessarily provide more increase in potency than homo-fusion. Nevertheless, because the use of SR31 does not conflict with homo-fusion owing to the non-competing

binding mode, SR31 may help further increase the potency of monoparatopic nanobodies that are already multivalent."

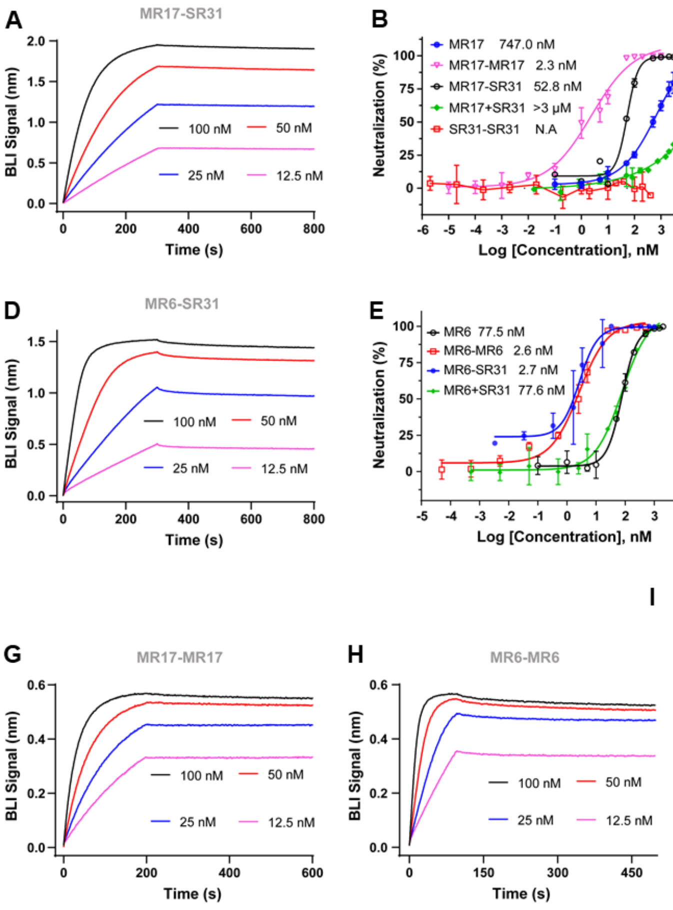

I

construct	K_D (nM)	k_{on} (M^{-1} s^{-1})	K_{off} (s^{-1})	IC_{50}, nM [μg mL^{-1}]
SR31	5.6	1.8×10^5	1.0×10^{-3}	N.A
SR31-SR31	0.7	1.6×10^5	1.1×10^{-4}	N.A
MR17	83.7	1.2×10^5	9.7×10^{-3}	747.0 [12.32]
MR17-SR31	0.3	1.3×10^5	3.8×10^{-5}	52.8 [1.65]
MR17 + SR31	N.D.	N.D.	N.D.	> 3,000
MR17-MR17	0.1	3.3×10^5	4.1×10^{-5}	2.3 [0.07]
MR6	23.2	3.1×10^5	7.2×10^{-3}	77.5 [1.32]
MR6-SR31	0.5	2.1×10^5	1.0×10^{-6}	2.7 [0.08]
MR6 + SR31	N.D.	N.D.	N.D.	77.6 [2.48]
MR6-MR6	0.2	7.9×10^5	1.6×10^{-4}	2.6 [0.08]
MR3	1.0	2.3×10^5	2.3×10^{-4}	24.4 [0.40]
MR3-SR31	N.D.	N.D.	N.D.	76.4 [2.38]

Fig. 6. SR31 increases binding affinity and neutralization activity of two fusion partners. (**A**) BLI binding assay with immobilized RBD and the biparatopic sybody MR17-SR31 as analyte at increasing concentrations (nM). (**B**) Neutralization assay of MR17 (blue), MR17-MR17 (magenta), MR17-SR31 (black), equimolar mix of MR17 and SR31 (MR17+SR31, green), and SR31-SR31 (red). (**C, D**) Binding kinetics for the RBD-binding by MR6 (**C**) or by MR6-SR31 (**D**). (**E**) Neutralization assay of MR6 (black), MR6-MR6 (red), MR6-SR31 (blue), and equimolar mix of MR6 and SR31 (MR6+SR31, green). (**F, G, H**) BLI binding assay with immobilized RBD and the monoparatopic divalent sybody SR31-SR31 (**F**), MR17-MR17 (**G**), or MR6-MR6 (**H**) as analyte at increasing concentrations (nM). (**I**) Summary of the comparison between monovalent sybodies, SR31-fusion (biparatopic) sybodies, homo-fusion (monoparatopic) sybodies for binding kinetics and neutralization activities. Binding kinetics and neutralization data for MR17 and MR3 are from reference (*35*). N.D., not determined; N.A., not applicable. In **A**, **C**, **D**, and **F-H**, concentrations (nM) of the sybodies used in the binding assay are labeled. In **B** and **E**, data are mean ± standard deviation from three independent experiments, and the x-axis indicates the concentration of the individual sybodies as opposed to the total sybody concentration.

The superior potency of MR17-MR17 over MR17-SR31 is discussed in the Discussion section:

"The reason for the superior neutralization activity of the homo-fusion constructs (MR17-MR17) over SR31-fusion constructs can be complex. First, one molecule of the homo-fusion sybody could potentially block two of the three RBMs when S assumes a 'two up-RBD' conformation (*58*). By contrast, the biparatopic SR31-fusion constructs could only act on one RBM, leaving two free RBMs that could bind the dimeric ACE2 much more efficiently compared to S proteins with one accessible RBM. In this sense, the superior potency of homo-fusion constructs is not surprising. Second, the potency of fusion constructs depends on, to some degree, the linker type and length, and the optimal linker for different constructs may differ. These factors make it difficult to directly compare different divalent types in a meaningful way. Third, as suggested by the neutralization assay with equimolar mixes of SR31 and MR17, the presence of SR31 may affect the recognition of RBM by MR17 in the context of trimeric S, despite they bind to the isolated RBD in a non-competing manner (**Fig. 5A, 5C**). Practically, because SR31 does not directly compete with MR17 or MR6 for epitope, it is not exclusive for RBM-binders regarding multivalent engineering. Rather, the gained potency by SR31 fusion on whatever existing form, if present, will be additive."

2. Most of the text and figures in this manuscript describe the crystal structure and characteristics of a non-neutralizing, non-RBD-binding sybody. Many crystal structures as well as high-resolution cryo-EM maps of antibody/nanobody-Spike/RBD complexes have already been published, with most of the antibodies/nanobodies having a neutralizing effect. It is unclear why so much attention has been put on the exact structural determination of SR31 while it does not possess extraordinary binding properties nor neutralizing capacity.

We have previously selected ~100 synthetic nanobodies from three libraries. Most of them are neutralizing nanobodies, some of which have been structurally, biochemically, and biologically characterized. This manuscript focuses on non-neutralizing nanobodies and we found that SR31 could be a useful tool to probe epitope, and to increase other nanobody's potency by fusion.

Indeed, crystal structures of the most important biparatopic constructs are missing. Addition of a crystal structure of the biparatopic sybody (SR31-MR17) will be very useful to this manuscript.

The crystal structure in Fig. 5 is indeed the complex for the biparatopic sybody (MR17-SR31) and RBD. Our apologies that the previous description was misleading. We have clarified this point in the revised manuscript:

"The neutral feature of SR31 so far suggests it could bind to RBD in addition to RBM binders such as MR17 and SR4 (*35*). Indeed, BLI assays showed no competition between SR31 and MR17 (**Fig. 5A**), indicating that RBD could bind both sybodies simultaneously. This non-

competing feature was also observed in the case of MR6 (**Fig. 5B**) which has been shown to neutralize the SARS-CoV-2 pseudovirus (*35*). As further proof for the simultaneous binding, we constructed a biparatopic sybody by fusing SR31 to the C-terminal of MR17 via a Gly-Ser linker with 34 amino acids (**Table S1**) and determined its structure in complex with RBD to 2.10 Å resolution (**Fig. 5C, Table 1**). This structure was similar to the individual MR17- and SR31-RBD complexes, with an overall Cα RMSD of 0.667 and 0.375 Å, respectively. Aligning the biparatopic sybody-RBD complex with the MR17-RBD structure revealed no appreciable changes at the MR17-binding surface (**Fig. 5C**), reinforcing the idea that SR31-binding does not allosterically change the RBM surface and that SR31 is highly likely to be compatible with RBM-binding antibodies."

3. When proposing new constructs as is being done in this manuscript, it is important to accurately place the results of the new constructs (biparatopic fusion constructs) into context and to accurately describe the molecular design of these constructs.

Thanks for the referee in catching up this. We have now cited Table S1 for the sequence information of the divalent constructs.

4. The authors have failed to sufficiently address the advantages of a biparatopic fusion construct in comparison to >10x more potent nanobodies, bivalent nanobodies (VHH-VHH) or monoclonal antibodies isolated from patients.

The advantage of biparatopic fusion is discussed as the following:

"This work generated two biparatopic sybodies, MR17-SR31 and MR6-SR31. Compared with monoparatopic divalent nanobodies or monoclonal antibodies, biparatopic nanobodies are more likely to be resistant to escape mutants because simultaneous mutations at two distinct and relatively remote epitopes should occur at a much lower rate than at a single epitope. Whether this is true for the biparatopic sybodies identified here remains to be tested. While the neutralizing activities of the biparatopic sybodies are comparable to some bivalent nanobodies and human VH domains in the literature (22, 36, 39, 52), we note the existence of a few ultra-potent nanobodies (*51*, *54*), especially those with high valency. Because SR31 does not compete with MR17 or MR6, one could construct hexavalent sybodies with three copies each of SR31 and MR17/MR6 to further increase potency. SR31 may also be fused to ultra-potent nanobodies in the literature to make even tighter fusion nanobodies, and to increase their size for longer *in vivo* half-lives (*29*) which is an important characteristic for nanobody drugs."

Minor comments

- The scientific English level of this manuscript is not sufficient for publication yet. I would suggest, if possible, to request a native speaker in the field to proofread the manuscript and correct the scientific English where necessary.

We have read the manuscript carefully, corrected several grammar errors, and reorganized some of the expressions in the manuscript.

- Line 91-95: FSEC: it is unclear how earlier retention of RBD suggests nanomolar affinity.

It was an inaccurate statement and we have removed it in the revised manuscript:

“In analytic fluorescence-detection size exclusion chromatography (FSEC), RBD eluted earlier in the presence of SR31 compared to RBD alone (**Fig. 1A**), suggesting the formation of a complex.”

- Fig. 2a-b: include arrows to indicate on which axis the rotation of the complex was performed.

Done.

- Line 130: also including the BSA of each of the CDRs would be very valuable.

Done.

- Line 148: another SARS-CoV-2/SARS-CoV neutralizing mAb that binds the CR3022 epitope has been identified recently, see https://www.ncbi.nlm.nih.gov/pmc/articles/PMC7418720 It will be useful to include in this manuscript.

We have discussed this interesting mAb at appropriate places, and modified Fig. 3B and 3C to reflect the changes.

“Comparing the epitopes of existing monoclonal antibodies showed that the SR31 epitope partly overlaps with CR3022 (*12*), and the recently identified EY6A (*22*) and COVA1-16 (*43*) (**Fig. 3B, 3C**). It has been established that the binding of the bulky CR3022/EY6A at the interface between RBD and the N-terminal domain (NTD) of the adjacent monomer destabilizes the S trimer and converts the pre-fusion conformation to the infection-incompetent post-fusion state, thus conferring neutralization activity (*21*, *22*). Despite the epitope overlapping, SR31 approaches RBD at a different angle to that of CR3022 (**Fig. 3C**). This angular difference, together with its minute size, may allow SR31 to bind two of the three sites in the ‘open’-S (*3*): the ‘up-RBD’, and the ‘down-RBD’ at the clockwise monomer (**Fig. 3D**) without destructing S. Similarly, despite epitope overlap (Fig. 3B), the binding of SR31 does not cause steric hindrance for ACE2-binding (**Fig. 3A**) as was the case for COVA1-16 (**Fig. 3C**) (*43*). Taken together, the structural data rationalize the high-affinity binding between SR31 and RBD, and its inability to neutralize SARS-CoV-2.”

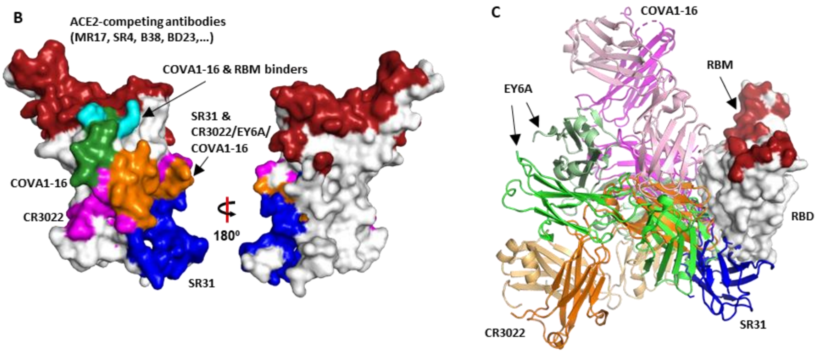

Fig. 3. SR31 engages RBD at a site distal to the receptor-binding motif. (**B**) Comparison of the SR31 epitope with epitopes for other RBD-targeting nanobodies (22, 35, 36, 39) and mAbs (13-15, 19, 20, 23, 24, 26-28). Red, the collective epitope of RBM-binders; blue, the SR31 epitope; magenta, the collective epitope of CR3022 and EY6A; green, the COVA1-16 epitope; cyan, the overlap between the epitopes of the RMB binders and COVA1-16; orange, the overlap between the CR3022/EY6A/COVA1-16 and SR31 epitope. (**C**) Comparison of the binding mode between SR31 (blue) and three mAbs including CR3022 (orange and wheat), EY6A (green and pale green), and COVA1-16 (pink and magenta). RBD is shown as white surface with RBM highlighted in red.

- The authors should aim to make the figure legend titles more descriptive of the message conveyed in that particular figure.

We thank the reviewer for the specific comment in improving our manuscript.

We have changed legends of Fig. 1, Fig. 3, and Fig. 4 to the following:

"Fig. 1. SR31 binds RBD with high affinity but does not perturb ACE2 binding.

Fig. 3. SR31 engages RBD at a site distal to the receptor-binding motif.

Fig. 4. SR31 causes dramatic structural arrangements of RBD at the binding site without distorting the receptor-binding motif."

- Line 159-164 do not belong in the results section and should be moved to introduction or discussion.

The previous manuscript had a combined section of Results & Discussion. We have now separated them in the revised manuscript.

- Line 213-214 and Fig. 4C: it would be helpful if the authors supply the reader with melting temperatures of a similarly-sized molecule before making comments that rigidity of the RBD

is a determining factor in its melting temperature. It could be that the melting temperature of more small, monomeric glycoproteins is around 95 C.

We thank the reviewer for the suggestion. The revised manuscript contains the following sentences:

"Remarkably, the dramatic rearrangements did not cause appreciable conformational changes of RBM (**Fig. 4A**) nor did it affect ACE2 binding (**Fig. 1D**). Given that RBD is a relatively small entity, and that the two surfaces are relatively close (~25 Å), this was somewhat unexpected. A probable explanation is that the core region of RBD is relatively rigid so that the RBM surface is not easily distorted allosterically. Because rigidity often correlates with heat stability (*44*), we measured the apparent melting temperature (T_m) of RBD using a fluorescence-detection size exclusion chromatography thermostability assay (*45*). Compared with glycoproteins with a similar size (erythropoietin, 21.3 kDa, T_m of 56 ℃ with 10-min heating; human granulocyte colony-stimulating factor, 21.2 kDa, T_m of 62.5 ℃ at a ramping rate of 1 ℃ min^{-1}; interferon beta, 22.3 kDa, T_m of ~70 ℃ at a ramping rate of 2 ℃ min^{-1}) (*46-48*), RBD (22.6 kDa) showed much higher heat stability, with an apparent T_m of greater than 95 ℃ (20-min heating) (**Fig. 4C**)."

- All the experiments in this paper are done with soluble RBD. It will be very useful to also include whole prefusion stabilized S protein in BLI assays or at least address this aspect in the discussion.

As suggested by Referee #1, we have performed a cell surface staining assay to show that SR31 binds to S expressed on the surface of HEK293T cells.

We also performed a pull-down assay to show that SR31 binds to S incorporated onto the pseudovirus.

We thank both reviewers for this comment to improve our manuscript.

- All neutralization experiments were performed on pseudovirus. Even though it is not necessary for a proof-of-concept study such as this, it would most certainly profit from addition of live SARS-CoV-2 virus neutralization experiments.

We can certainly appreciate the importance of live virus in neutralization antibody studies. However, because the access to BSL3 laboratories is extremely difficult in all over China and we (the two groups) unfortunately do not have resources to conduct such experiments.
