# Supplementary material for: A high-affinity RBD-targeting nanobody improves fusion partner’s potency against SARS-CoV-2
Source: PLoS Pathog. 2021 Mar 3;17(3):e1009328. doi: 10.1371/journal.ppat.1009328 (PMC7959386; doi:10.1371/journal.ppat.1009328)
Supplement: Peer review history

# Peer review: Decision Letter 0

**DOI:** 10.1371/journal.ppat.1009328.r001

**Authors:** Ron A. M. Fouchier

8 Oct 2020

Dear Dr. Li,

Thank you very much for submitting your manuscript "A high-affinity RBD-targeting nanobody improves fusion partner’s potency against SARS-CoV-2" for consideration at PLOS Pathogens. As with all papers reviewed by the journal, your manuscript was reviewed by members of the editorial board and by several independent reviewers. In light of the reviews (below this email), we would like to invite the resubmission of a significantly-revised version that takes into account the reviewers' comments.

We cannot make any decision about publication until we have seen the revised manuscript and your response to the reviewers' comments. Your revised manuscript is also likely to be sent to reviewers for further evaluation.

When you are ready to resubmit, please upload the following:

[1] A letter containing a detailed list of your responses to the review comments and a description of the changes you have made in the manuscript. Please note while forming your response, if your article is accepted, you may have the opportunity to make the peer review history publicly available. The record will include editor decision letters (with reviews) and your responses to reviewer comments. If eligible, we will contact you to opt in or out.

[2] Two versions of the revised manuscript: one with either highlights or tracked changes denoting where the text has been changed; the other a clean version (uploaded as the manuscript file).

Important additional instructions are given below your reviewer comments.

Please prepare and submit your revised manuscript within 60 days. If you anticipate any delay, please let us know the expected resubmission date by replying to this email. Please note that revised manuscripts received after the 60-day due date may require evaluation and peer review similar to newly submitted manuscripts.

Thank you again for your submission. We hope that our editorial process has been constructive so far, and we welcome your feedback at any time. Please don't hesitate to contact us if you have any questions or comments.

Sincerely,

Ron A. M. Fouchier, Ph.D.

Section Editor

PLOS Pathogens

Ron Fouchier

Section Editor

PLOS Pathogens

Kasturi Haldar

Editor-in-Chief

PLOS Pathogens

​orcid.org/0000-0001-5065-158X

Michael Malim

Editor-in-Chief

PLOS Pathogens

orcid.org/0000-0002-7699-2064

***********************

Reviewer's Responses to Questions

**Part I - Summary**

Please use this section to discuss strengths/weaknesses of study, novelty/significance, general execution and scholarship.

Reviewer #1: The authors report the isolation and characterization of nanobody SR31 derived from a synthetic library by ribosome display using SARS-CoV-2 RBD as the target. SR31 can bind to SARS-CoV-2 RBD, does not compete for hACE2 binding and does not neutralize SARS-CoV-2. The co-crystal structure of SR31 with RBD was determined, which revealed a hydrophobic interface and a large epitope. SR31 and RBD undergo an induced fit upon binding. The structures of SR32 fused to nanobody MR17 and MR6, in complex with the RBD were also determined. The pseudotype virus neutralizing activity of MR17 and MR6 increased 27-40 fold when merged with SR31. Complete neutralization was achieved with MR6 but not with SR31-MR6.

This is an interesting, in general well performed study.

Reviewer #2: In this manuscript, the authors report several crystal structures of nanobodies that bind to the receptor binding domain (RBD) or receptor binding motif (RBM) of SARS-CoV-2 spike. They have also characterized fusion constructs of synthetic nanobodies (sybodies) and found that biparatopic fusion of two nanobodies targeting different epitopes on the RBD increases their affinity and neutralization potency. While this work certainly sheds additional light on the already vast body of work on nanobodies in the context of SARS-CoV-2, there are several issues that need to be addressed before publication can be recommended.

**********

**Part II – Major Issues: Key Experiments Required for Acceptance**

Please use this section to detail the key new experiments or modifications of existing experiments that should be absolutely required to validate study conclusions.

Generally, there should be no more than 3 such required experiments or major modifications for a "Major Revision" recommendation. If more than 3 experiments are necessary to validate the study conclusions, then you are encouraged to recommend "Reject".

Reviewer #1: Major remarks:

1. The authors should show that SR31 can bind to full length spike, either as a purified recombinant protein or when expressed on the surface of transfected cells. Such an experiment can demonstrate that the “greasy” epitope is accessible in full length spike.

2. It is important to show that the enhanced neutralizing activity of SR31-MR17 and SR31-MR6 is also or not observed when the respective nanobodies are simply mixed at equimolar concentrations in the neutralization assays.

Reviewer #2: Major comments

- The main issue is the lack of many essential controls in the most important figure of the manuscript, Figure 6. For many SARS-CoV-2 antibodies and nanobodies, avidity effects have been described to contribute quite heavily to neutralization potency and binding (https://www.ncbi.nlm.nih.gov/pmc/articles/PMC7418720/). Importantly, when describing the sybody fusion construct SR31-MR17, an essential comparison would be to compare it to SR31-SR31 and MR17-MR17 fusion constructs to be able to attribute the gain of affinity and neutralization capacity to the biparatopic nature of the construct and not simply because of increase of avidity. The same is true for Figure 6C-D-E-F. Especially since the predominant binding data is in the form of BLI and the density of the RBD on the biosensor will be quite high, it is very relevant and important to address bivalent monoparatopic constructs (SR31-SR31, MR17-MR17 etc.).

- Most of the text and figures in this manuscript describe the crystal structure and characteristics of a non-neutralizing, non-RBD-binding sybody. Many crystal structures as well as high-resolution cryo-EM maps of antibody/nanobody-Spike/RBD complexes have already been published, with most of the antibodies/nanobodies having a neutralizing effect. It is unclear why so much attention has been put on the exact structural determination of SR31 while it does not possess extraordinary binding properties nor neutralizing capacity. Indeed, crystal structures of the most important biparatopic constructs are missing. Addition of a crystal structure of the biparatopic sybody (SR31-MR17) will be very useful to this manuscript.

- When proposing new constructs as is being done in this manuscript, it is important to accurately place the results of the new constructs (biparatopic fusion constructs) into context and to accurately describe the molecular design of these constructs. The authors have failed to sufficiently address the advantages of a biparatopic fusion construct in comparison to >10x more potent nanobodies, bivalent nanobodies (VHH-VHH) or monoclonal antibodies isolated from patients.

**********

**Part III – Minor Issues: Editorial and Data Presentation Modifications**

Please use this section for editorial suggestions as well as relatively minor modifications of existing data that would enhance clarity.

Reviewer #1: Other remarks:

1. Line 57: “nanobodies are known to cause little immunogenicity” is a too strong statement. There is only one nanobody-based biologic on the market for clinical use. Anti-drug antibodies against Alx-0171, a homotrivalent nanobody construct, have been reported in infants that were hospitalized due to RSV and were enrolled in phase 2 and 3 trials with this drug. “Nanobodies are considered to be poorly immunogenic in human” would be a more prudent statement.

2. Line 88: most of the sybody binders should be “most of the RBD binders”.

3. Line 93: “caused earlier retention” should be “eluted earlier”.

4. Line 142: the nanobody against SARS-CoV-1 and -2 that is described in ref 34, does not bind to the RBM yet it competes with hACE2 and can neutralize SARS-CoV-2. Please correct the statement.

Reviewer #2: Minor comments

- The scientific English level of this manuscript is not sufficient for publication yet. I would suggest, if possible, to request a native speaker in the field to proofread the manuscript and correct the scientific English where necessary.

- Line 91-95: FSEC: it is unclear how earlier retention of RBD suggests nanomolar affinity.

- Fig. 2a-b: include arrows to indicate on which axis the rotation of the complex was performed.

- Line 130: also including the BSA of each of the CDRs would be very valuable.

- Line 148: another SARS-CoV-2/SARS-CoV neutralizing mAb that binds the CR3022 epitope has been identified recently, see https://www.ncbi.nlm.nih.gov/pmc/articles/PMC7418720/. It will be useful to include in this manuscript.

- The authors should aim to make the figure legend titles more descriptive of the message conveyed in that particular figure.

- Line 159-164 do not belong in the results section and should be moved to introduction or discussion.

- Line 213-214 and Fig. 4C: it would be helpful if the authors supply the reader with melting temperatures of a similarly-sized molecule before making comments that rigidity of the RBD is a determining factor in its melting temperature. It could be that the melting temperature of more small, monomeric glycoproteins is around 95 C.

- All the experiments in this paper are done with soluble RBD. It will be very useful to also include whole prefusion stabilized S protein in BLI assays or at least address this aspect in the discussion.

- All neutralization experiments were performed on pseudovirus. Even though it is not necessary for a proof-of-concept study such as this, it would most certainly profit from addition of live SARS-CoV-2 virus neutralization experiments.

**********

PLOS authors have the option to publish the peer review history of their article (what does this mean?). If published, this will include your full peer review and any attached files.

If you choose “no”, your identity will remain anonymous but your review may still be made public.

**Do you want your identity to be public for this peer review?** For information about this choice, including consent withdrawal, please see our Privacy Policy.

Reviewer #1: No

Reviewer #2: No

Figure Files:

While revising your submission, please upload your figure files to the Preflight Analysis and Conversion Engine (PACE) digital diagnostic tool, https://pacev2.apexcovantage.com. PACE helps ensure that figures meet PLOS requirements. To use PACE, you must first register as a user. Then, login and navigate to the UPLOAD tab, where you will find detailed instructions on how to use the tool. If you encounter any issues or have any questions when using PACE, please email us at figures@plos.org.

Data Requirements:

Please note that, as a condition of publication, PLOS' data policy requires that you make available all data used to draw the conclusions outlined in your manuscript. Data must be deposited in an appropriate repository, included within the body of the manuscript, or uploaded as supporting information. This includes all numerical values that were used to generate graphs, histograms etc.. For an example see here on PLOS Biology: http://www.plosbiology.org/article/info%3Adoi%2F10.1371%2Fjournal.pbio.1001908#s5.

Reproducibility:

To enhance the reproducibility of your results, PLOS recommends that you deposit laboratory protocols in protocols.io, where a protocol can be assigned its own identifier (DOI) such that it can be cited independently in the future. For instructions see http://journals.plos.org/plospathogens/s/submission-guidelines#loc-materials-and-methods

# Author comment: Author response to Decision Letter 0

**DOI:** 10.1371/journal.ppat.1009328.r002

21 Dec 2020

Attachment Submitted filename: Response_letter_DL_20201218.pdf Click here for additional data file.

# Peer review: Decision Letter 1

**DOI:** 10.1371/journal.ppat.1009328.r003

**Authors:** Ron A. M. Fouchier

21 Jan 2021

Dear Dr. Li,

We are pleased to inform you that your manuscript 'A high-affinity RBD-targeting nanobody improves fusion partner’s potency against SARS-CoV-2' has been provisionally accepted for publication in PLOS Pathogens.

Before your manuscript can be formally accepted you will need to complete some formatting changes, which you will receive in a follow up email. A member of our team will be in touch with a set of requests.

Please note that your manuscript will not be scheduled for publication until you have made the required changes, so a swift response is appreciated.

IMPORTANT: The editorial review process is now complete. PLOS will only permit corrections to spelling, formatting or significant scientific errors from this point onwards. Requests for major changes, or any which affect the scientific understanding of your work, will cause delays to the publication date of your manuscript.

Should you, your institution's press office or the journal office choose to press release your paper, you will automatically be opted out of early publication. We ask that you notify us now if you or your institution is planning to press release the article. All press must be co-ordinated with PLOS.

Thank you again for supporting Open Access publishing; we are looking forward to publishing your work in PLOS Pathogens.

Best regards,

Ron A. M. Fouchier, Ph.D.

Section Editor

PLOS Pathogens

Ron Fouchier

Section Editor

PLOS Pathogens

Kasturi Haldar

Editor-in-Chief

PLOS Pathogens

​orcid.org/0000-0001-5065-158X

Michael Malim

Editor-in-Chief

PLOS Pathogens

orcid.org/0000-0002-7699-2064

***********************************************************

Reviewer Comments (if any, and for reference):

Reviewer's Responses to Questions

**Part I - Summary**

Please use this section to discuss strengths/weaknesses of study, novelty/significance, general execution and scholarship.

Reviewer #2: The authors have adequately addressed my concerns. The new data showing that the biparatopic MR6-SR32 sybody is not more potent than the MR6-MR6 control (newly added data) is somewhat disappointing as it leaves one to wonder what the practical advantage is the biparatopic construct. Nevertheless, it is intriguing that the non-neutralizing component SR32 confers an unusual increase neutralization potency to the biparatopic construct. Avidity is likely to play am important role, but is probably not the entire story.

**********

**Part II – Major Issues: Key Experiments Required for Acceptance**

Please use this section to detail the key new experiments or modifications of existing experiments that should be absolutely required to validate study conclusions.

Generally, there should be no more than 3 such required experiments or major modifications for a "Major Revision" recommendation. If more than 3 experiments are necessary to validate the study conclusions, then you are encouraged to recommend "Reject".

Reviewer #2: (No Response)

**********

**Part III – Minor Issues: Editorial and Data Presentation Modifications**

Please use this section for editorial suggestions as well as relatively minor modifications of existing data that would enhance clarity.

Reviewer #2: (No Response)

**********

PLOS authors have the option to publish the peer review history of their article (what does this mean?). If published, this will include your full peer review and any attached files.

If you choose “no”, your identity will remain anonymous but your review may still be made public.

**Do you want your identity to be public for this peer review?** For information about this choice, including consent withdrawal, please see our Privacy Policy.

Reviewer #2: No

# Editor report: Acceptance letter

**DOI:** 10.1371/journal.ppat.1009328.r004

**Authors:** Ron A. M. Fouchier

18 Feb 2021

Dear Dr. Li,

We are delighted to inform you that your manuscript, "A high-affinity RBD-targeting nanobody improves fusion partner’s potency against SARS-CoV-2," has been formally accepted for publication in PLOS Pathogens.

We have now passed your article onto the PLOS Production Department who will complete the rest of the pre-publication process. All authors will receive a confirmation email upon publication.

The corresponding author will soon be receiving a typeset proof for review, to ensure errors have not been introduced during production. Please review the PDF proof of your manuscript carefully, as this is the last chance to correct any scientific or type-setting errors. Please note that major changes, or those which affect the scientific understanding of the work, will likely cause delays to the publication date of your manuscript. Note: Proofs for Front Matter articles (Pearls, Reviews, Opinions, etc...) are generated on a different schedule and may not be made available as quickly.

Soon after your final files are uploaded, the early version of your manuscript, if you opted to have an early version of your article, will be published online. The date of the early version will be your article's publication date. The final article will be published to the same URL, and all versions of the paper will be accessible to readers.

Thank you again for supporting open-access publishing; we are looking forward to publishing your work in PLOS Pathogens. 

Best regards,

Kasturi Haldar

Editor-in-Chief

PLOS Pathogens

​orcid.org/0000-0001-5065-158X

Michael Malim

Editor-in-Chief

PLOS Pathogens

orcid.org/0000-0002-7699-2064
